# E3 ubiquitin ligase TRIM21 targets TIF1γ to regulate β-catenin signaling in glioblastoma

**DOI:** 10.7150/thno.85662

**Published:** 2023-09-04

**Authors:** YanLan Li, Lingbo Bao, Hong Zheng, Mingying Geng, TianYi Chen, Xiaoyan Dai, He Xiao, Lujie Yang, Chengyi Mao, Yuan Qiu, Yu Xu, Dong Wang, Meng Xia Li, Qian Chen

**Affiliations:** 1 Cancer Center of Daping Hospital, Army Medical University, Chongqing 400037, China.; 2 Chongqing University Cancer Hospital, Chongqing, China.; 3 Department of Thoracic Surgery, Xinqiao Hospital, Third Military Medical University (Army Medical University), Chongqing 400037, China.; 4 The Pathology of Daping Hospital Army Medical University, Chongqing 400037, China.; 5 Department of General Surgery, Xinqiao Hospital, Third Military Medical University (Army Medical University), Chongqing 400037, China.

**Keywords:** TRIM21, β-catenin, TRIMs family, TIF1γ, ubiquitination

## Abstract

**Background:** Elucidation of the mechanism of ubiquitation has led to novel ways to treat glioblastoma (GBM). A tripartite motif (TRIM) protein mediates a reversible, stringent ubiquitation which is closely related to glioma malignancy. This study intends to screen the most vital and abnormal regulating component of the tripartite motif protein and to explore its underlying mechanisms.

**Methods:** TRIM21 is identified as an important oncogene that accelerates the progression of glioma cell through database in a multidimensional way and this is confirmed in human samples and cells. Tandem Mass Tags (TMT) and MS analysis are performed to discover the substrates of TRIM21.The underlying mechanisms are further investigated by CO-IP, luciferase reporter assays and gain and loss of function assays. In vivo treatment with siRNA is applied to evaluate the therapeutic significance of TRIM21.

**Result:** We screened a panel of TRIM proteins and identified TRIM21, a E3 ubiquitin-protein ligase and autoantigen, as well as a prognostic biomarker for GBM. Functionally, high expression of wild-type TRIM21 accelerates tumor progression *in vitro* and *in vivo*, whereas TRIM21 mutants, including one with a critical RING-finger deletion, do not. Mechanistically, TRIM21 stimulates K63-linked ubiquitination and subcellular translocation of active β-catenin from the cytoplasm to the nucleus. Moreover, TRIM21 forms a complex with the β-catenin upstream regulator, TIF1γ, in the nucleus and accelerated its degradation by inducing K48-linked ubiquitination at K5 site, consequently increasing further nuclear β-catenin presence. Endogenous TRIM21 levels are found to be inversely correlated with TIF1γ but positively correlated with β-catenin in glioma tissue microarray experiments. Furthermore, direct injection of TRIM21 small interfering RNA (siRNA) into U87 cell-derived tumors (in vivo treatment with siRNA) is proved to inhibit tumor growth in nude mice.

**Conclusion:** This work suggests that TRIM21/TIF1γ/β-catenin axis is involved in the progression of human GBM. TRIM21 is a promising therapeutic and prognostic biomarker for glioma with hyperactive β-catenin.

## Introduction

GBM is prevalent in adult brain cancer, characterized by its rapid growth, invasiveness and poor survival 1. A combination of oncogenic gain of function and tumor-suppressive loss of function contributes to GBM progression. Growing evidence indicates that the ubiquitin-proteasome system controls the fate of oncogenic and tumor suppressor proteins via E3 ubiquitin ligases, which has a profound influence on glioma malignancy 2.

The tripartite-motif (TRIM) protein family contains approximately 80 members and represents one of the largest collections of E3 ubiquitin ligases. Structurally, these proteins featured an N-terminal TRIM motif that includes one RING-finger domain, one or two B box motifs, and a coiled-coil region 3, 4. Numerous studies revealed that TRIM proteins play various roles in cell biology, including in innate immunity or anti-tumor immune response 5, 6, oncogenic or anti-oncogenic development 7, chromatin modification 8, pyroptosis 9 and self-renewal in cancer stem cells 10, via controlling gene transcription, post-translational modifications, and interaction with pathogens. Genetic alterations in *TRIM* family members frequently emerge in many tumor types, ultimately affecting various tumor properties 11. Nevertheless, the precise role and mechanisms of TRIM proteins in GBM remain undefined.

The canonical Wnt signaling pathway often remains activated in GBM 12. Once bound by Wnt, Frizzled/LDL-recepor-related protein promotes translocation of β‐catenin from the cytoplasm to the nucleus, subsequently activating T‐cell factor (TCF)/lymphoid enhancer binding factor (LEF), which elicits the expression of Wnt target genes such as C‐Myc and Cyclin D1. In the absence of Wnt, cytoplasmic β‐catenin is phosphorylated by GSK3β and CK1, leading to E3 ligase β-TrCP mediated ubiquitination 13. The tripartite motif (TRIM) proteins, act as key regulators in the development of diverse cancers due to modulating transcriptional activity of Wnt/β‐catenin. For instance, TRIM50 prevents cell proliferation and invasion via degrading β‐catenin in Gastric cancer. TIF1γ, also named TRIM33, interacts with and degrades phosphorylated β‐catenin via inhibiting its downstream β‐catenin-dependent cascade in glioma 14.

Of the TRIM proteins, TRIM21 is identified as a prognostic biomarker and a regulator of malignancy for GBM using public databases and clinical glioma specimens. Depending on the cancer type and the carcinogenesis effector, TRIM21 may change cancer progression, alternatively through increasing the ubiquitination of oncogenic or tumor suppressor proteins 15.We also discover that TRIM21 not only promotes K63-linked ubiquitination of β‐catenin, accelerating its translocation into nuclei, but also increases β‐catenin in nucleus by enhancing K48-linked ubiquitination of TIF1γ, another important regulator of β‐catenin 14, 16, thereby resulting in high transcriptional activity of Wnt/β‐catenin in GBM cells. Furthermore, we reveal the significance of a new mutant R443W of TRIM21, evaluate the antitumor activity using si-TRIM21 and anticipate that targeting TRIM21 may be a potential therapeutic strategy for GBM.

## Materials and Methods

### Reagents and Antibodies

Recombinant human Wnt3a and Ubiquitin inhibitor MG132 were purchased from Med-Chem-Express (New Jersey, USA). Cycloheximide (CHX) was got from Sigma-Aldrich (St Louis, MO, USA). Anti-TRIM21 antibody (67136-1-Ig), Anti-C-MYC antibody (10828-1-AP) and Anti-GAPDH antibodies (60004-1-Ig) were acquired from Proteintech. Anti-HA antibody (#3724), Anti-TIF1γ antibody (#90051), Anti-Flag antibody (#2368), Anti-β-catenin antibody (#8480) and Anti-HIS (#8761) antibodies were obtained from Cell Signaling Technology (Danvers, USA), and Anti-Ki-67 (ab92742) antibody was got from Abcam. (Cambridge, UK).

### Patients and tumor specimens

All glioma tissue microarrays were purchased from Biochip (Shanghai, China), which were collected from 2008 to 2011. All patients were followed up for 5 years and clinic pathologic parameters were shown in Table S1. Nonneoplastic brain tissue samples (NBT; n=5) were obtained from the Department of Pathology at Daping Hospital. The study concerning human specimens were performed in accordance with the principles of the Helsinki Declaration and approved by the Ethics Committee of DaPing hospital, Third Military Medical University. The clinic pathological information and TRIM family expression were downloaded from The Cancer Genome Atlas TCGA and Rembrandt dataset.

### Bioinformatic analyses of human GBMs from database

The gene expression in human GBMs with follow-up information was analyzed using gene profiling data (AffyU133a, AgilentG4502A, IlluminaHiSeq, GBMLGG dataset) from the TCGA database and the Rembrandt database (http://gliovis.bioinfo.cnio.es/).

For the GEO database (GSE108474), TRIM21 expression and positively regulative Wnt signalling pathway score were calculated by GSTA algorithm. The DEG functional analyses were performed using the clusterProfiler and ggplot2 R packages, with P < 0.05 considered statistically significant.

### IHC staining and scoring

The tissue microarray was deparaffinized, hydrated and incubated with rabbit monoclonal anti-TRIM21 (Proteintech,1: 200) and rabbit polyclonal anti-TIF1γ (CST, 1:200), anti-β-catenin (CST, 1:500), anti-IDH1-R132H (MAB-#0062) antibodies respectively at 4 °C overnight, followed by subsequent incubation for 1 h with goat anti-rabbit IgG-HRP for detection. DAB (Dako, Santa Clara, CA, USA) was used as a chromogen. We followed a previously described protocol to quantify staining intensity 17. The tissue microarray was evaluated by two double-blinded pathologists. Final scores of TRIM21 and TIF1γ as well as β-catenin were obtained by multiplying the strength score by the distribution score. The cut-off value of the IHC score was determined by relative risk analysis with the statistical software X-tile 18. The cutoff score in various analyses was 3 for both anti-β-catenin and anti-TRIM21 staining, whereas the cutoff score was 1 for both anti-TIF1γ respectively.

### Cell culture

Primary GBM cells (GBM-1) were isolated from human GBM specimens as previously described 17. GBM cell lines (U87-MG, U251-MG)) and HEK-293T were obtained from the ATCC. It was maintained in DMEM (HyClone, Logan, Utah, USA) containing 10% FBS (PAN, South America) and maintained at 37 °C in a humidified chamber containing 5% CO2.

### Constructs, siRNA, lentivirus for transfection

Plasmid constructs for ectopic expression of full-length TRIM21 pcDNA3.1-HIS-TRIM21-FL; a RING-finger domain deletion mutant of TRIM21 (pcDNA3.1-HIS-TRIM21-ΔRING) and other TRIM21-mutants were purchased from Sino Biological (Beijing). Additionally, the expression vectors encoding pcDNA3.1-FLAG-TIF1γ, and pcDNA3.1-FLAG-TIF1γ-mutants (K5R, K1115R), other FLAG-TIF1γ- domain deletion, pcDNA3.1-β-catenin, and pcDNA3.1-S33Y-β-catenin, pcDNA3.1-K354R-β-catenin, other β-catenin - domain deletion mutants HA-UB, His-UB and His-UB-mutants were also generated and purchased from Sino Biological (Beijing, China), SMARTpool siRNA duplexes specific for TIF1γ, LAMC2 and a nontargeting siRNA (siControl) were purchased from RiboBio. The sequences of siRNA were as follows:

siTIF1γ-1 GGAAGATGCTGGCTAAGT; siTIF1γ-2: GCCAGATATTCCACCATA; siLAMC1-1: GCAAGCGAGTGTATGAAGA; siLAMC1-2: GCAAGCAG-GTGTTGTTGAGTTA. siTRIM21: 5'-GAUGGUGUCUGCUAUUGUATT-3'. HEK 293T, U87-MG and U251-MG cells were transfected with siRNA or plasmid using Lipo 3000 (Thermofisher, California, USA).

For overexpression of TRIM21-HA, a R443W mutant of TRIM21-HA, TIF1γ-Flag in GBM cells, the TRIM21-HA, TRIM21^R443W^-HA, TIF1γ-Flag cDNA was cloned into pLOV-CMV-eGFP-EF1a-PuroR lentiviral vector (Genechem, Shanghai, China). Lentiviral particles containing TRIM21-HA and TIF1γ-Flag were packaged by Helper 1.0. Cells with stable TRIM21 or/and TIF1γ. To generate stable TRIM21 knockdown GBM cells, U87-MG and GBM-1 cells (1×10^6^ /well) were transfected with lentiviral vector (Genechem, Shanghai, China) carrying self-complementary hairpin DNA fragments that could generate TRIM21-specific shRNA, or scrambled RNA (scramble). The list of shRNA was shown as follows: Scramble: 5'-TTCTCCGAACGTGTCACGT-3'; shTRIM21-1: 5'-TGGCATGGTCTCCTTCTACAA-3'; shTRIM21-2: 5'-GAGTTGGCTGAGAAGTT'GGAA-3'; shTRIM21-3: 5'-CTGCCTTCTTTATGGGACTTA-3'. The cells were treated overnight with the medium containing retrovirus or lentivirus harvested 72 h after transfection. Selection of stable clones was carried out using puromycin.

### Nuclear extraction

Cytoplasmic and nuclear fractions were extracted from cells using a Minute™ Cytoplasmic and Nuclear Extraction Kit (Beyotime, Shanghai, China), according to the manufacturer's instructions.

### Quantitative real-time reverse-transcription PCR

qPCR was conducted as the previous study. The sequences of the PCR primers were as follows: β-catenin forward, 5'-AGGAATGAAGGTGTGGCGACA-3'; β-catenin reverse, 5'-TGGCAGCCCATCAACTGGAT-3'; GAPDH forward, 5-AATCCCATCA CCATCTTCCA-3; GAPDH reverse, 5'-TGGACTCCACGACGTACTCA-3'; TIF1γ Forward 5'-AGCAACGGCGACATCCA-3; TIF1γ reverse 5'-CCAGCCCTCCTAGAGC -3'.

### Immunoblotting and immunoprecipitation

For immunoblotting, the cells were lysed with 300 μL RIPA lysis buffer containing a protease and phosphatase inhibitor cocktail (Thermo Fisher) at various time points and protein concentrations were detected using the BCA Protein Assay Kit (Beyotime, Shanghai, China). Total proteins (30 μg equivalent) were then separated by 8% or 10% SDS-PAGE (Beyotime, Shanghai, China), transferred using a 0.22-μm polyvinylidene fluoride (PVDF) filter (Millipore) and blocked with blocking solution (Beyotime, Shanghai, China). The PVDF filter was incubated with 1:1000 rabbit anti-TRIM21 (Proteintech), 1:1000 rabbit anti-TIF1γ (Cell Signaling Technology), 1:1000 rabbit anti-β-catenin (Cell Signaling Technology), 1:1000 rabbit anti-Cmyc (Proteintech), 1:1000 rabbit anti-Flag (Cell Signaling Technology), 1:1000 rabbit anti-HA (Cell Signaling Technology), 1:1000 rabbit anti-HIS (Cell Signaling Technology), and 1:1000 mouse anti-GAPDH (Proteintech) overnight at 4 °C. After washing with TBST, membranes were incubated with appropriate HRP-conjugated secondary antibodies (1:10000) for 1 h at the room temperature, and then washed with TBST three times and visualized by chemiluminescent detection, then the densitometric values were determined by a gel image analysis system (Bio-Rad, Hercules, CA, USA).

For immunoprecipitation, cells were grown in 6-well plates and transfected with the appropriate plasmids, which was extracted by cell lysis buffer for western and IP (Beyotime, Shanghai, China) with protease inhibitors. IP lysates was were captured with the appropriate anti-Flag M2 magnetic beads (M8823, Sigma-Aldrich), TIF1γ for 1 h at 4°C according to the manufacturer's instructions. After washing three times with equilibrium buffer (50 mM Tris HCl, 150 mM NaCl, pH 7.4), the beads were boiled with SDS-PAGE loading buffer for 10 min. The total cell lysates (input) were loaded and separated by 10% SDS-PAGE, after that, the immunoprecipitates were eluted, neutralized, and SDS degenerated for further immunoblotting.

### In vivo Ubiquitination assay

To detect TIF1γ or β-catenin ubiquitination, 293T cells or U87-MG cells were transfected with HA-Ub (ubiquitin) or HA-Ub mutants and indicated vectors, pretreated with MG132 for 4 h. Whole-cell lysates were collected using by (50 mM Tris-HCl, pH 7.5, 0.5 mM EDTA, 1 mM DTT, 1% SDS, and a protease inhibitor) and incubated at 95 °C for 5 min. After that, 900 μl Tris-HCl buffer (50 mM, pH 7.5) were added, sonicated. and centrifugated, the cell lysates were subjected to immunoprecipitation.

### In vitro Ubiquitination assay

In vitro ubiquitination of β-catenin was performed at 37 °C for 60 min in 50 μl reaction buffer (50 mM Tris pH 7.5, 5 mM MgCl_2_ and 2 mM DTT), containing purified Flag-β-catenin, UBE1 (100 nM, Boston Biochem), UbcH13/Uev1a (1 μΜ, Boston Biochem), His-ubiquitin and TRIM21 protein or IgG. The reaction mixtures were heated and then diluted with buffer (0.05% Triton X-100, 0.1M Na_2_HPO_4_ /NaH_2_PO_4_, 10 mM imidazole, pH 8.0) For the purification of ubiquitinated β-catenin using Ni-NTA beads (Qiagen). The eluted proteins were analysed by immunoblotting.

### Tandem Mass Tags (TMT) and MS analysis

For Tandem Mass Tags (TMT), TMT labeling was performed according to manufacturer's instructions 19. Briefly, peptides were reconstituted in TMT reagent buffer and labeled with different TMT labeling reagents. The internal reference sample pooled from all the mocks and OE*TRIM21* samples was labeled using channel 126 for each batch of TMT labeling experiment allowing comparison of relative protein abundance across different TMT experiments. The labeled samples were then mixed and subjected to Sep-Pak C18 desalting. The labeling efficiency of each labeled mixture was examined by mass spec identification of 2 μg of the mixture with TMT (N-terminal/K) as variable modifications. The remaining mixture for each group of TMT experiment was fractionated using high pH reverse phase chromatography into 60 fractions and further concatenated into 20 fractions. Each fraction was vacuum-dried and stored at -80 °C until MS analysis. For the liquid chromatography-MS analysis, the gel pieces with TRIM21-FLAG immunoprecipitated complex or control were dehydrated in acetonitrile, dried in a speed vacuum and digested with trypsin. The peptides were extracted from the polyacrylamide and were evaporated for MS analysis using LTQ-Orbitrap Elite Mass Spectrometer System (Thermo Scientific, Waltham, MA, USA). TMT and MS analysis was finished by Jinkairui Biological Engineering company in WuHan. GO pathway enrichment analysis were performed using Dr. Tom, an online analysis system of BGI. The images of Venn diagram, and heatmap plots were performed using the OmicStudio tools at an open website (https://www.omicstudio.cn). Fold change > 2.0 and p < 0.05 were the cut off.

### GST Pull-down assay

The plasmids for GST-His-TIF1γ and His-tagged β-catenin were transfected into E. coli. Crude bacterial lysates were then prepared by sonication in ice-cold PBS in the presence of a protease inhibitor mixture. The in vitro transcription and translation experiments were performed with rabbit reticulocyte lysate (TNT Systems; Promega). Approximately 100 µg GST- His-TIF1γ fusion protein was immobilized in 50 µL of glutathione agarose and equilibrated before being incubatedtogether at 4 °C for 60 min with gentle rocking motion. His-tagged β-catenin and/or His-tagged TRIM21 (MCE HY-P71791) were added to the immobilized GST-His-TIF1γ and incubated overnight at 4 °C under gentle rotation. The pull-down samples were analysed by immunoblotting.

### Promoter reporters and dual-luciferase assay

U87-MG cells were transfected with the TOP-FLASH or FOP-FLASH reporter plasmids together with pRL-TK (Beyotime) using lipo-3000 respectively for 24 h. Luciferase activity was measured with the Dual-Luciferases Reporter Assay kit (Beyotime) according to manufacturer's protocols after transfection. It was detected using the Dual-Luciferase® Reporter Assay System (Promega, Madison, USA).

### Brdu assay and CCK-8 assay

The growth of GBM cells were measured using BeyoClick™ EdU Cell Proliferation Kit with TMB (C008S, Beyotime), according to the manufacturer's instructions. Cells were then examined and counted under Fluorescence microscope.

For CCK8 assay, 1×10^3^ cells per well were cultured in 96-well plates and then detected using Cell Counting Kit-8 (C0037, Beyotime) reagent by measuring the absorbance at 450 nm.

### Wound-healing assay and transwell invasion assay

A wound-healing assay and transwell Invasion assay were carried to examine the mobility and invasion of cancer cell respectively, as previously described 20. For wound-healing assay, the migratory ability of the cells was presented as the gap distance recovered compared with the original gap. For transwell Invasion assay, the invasion ability of cells was counted in five randomly selected high-powered fields under a microscope.

### IF analysis

Cells were fixed with 4% buffered paraformaldehyde and permeabilized with 0.5% Triton X-100 for 3 min. Cells were incubated with the primary antibody overnight at 4 °C and then incubated with fluorescent secondary antibodies and DAPI at 37 °C Images were observed using a confocal microscope (LSM780NLO, Zeiss, Germany).

### Animal experiment

U87-MG (1×10^5^) with pLVX -linker-luciferase lentivirus transfected were injected intracranially into the frontal lobes of 5-week-oldfemale NOD-SCID mice (n = 5 per group) as previously described 21. The xenograft tumors were detected and quantified by bioluminescence imaging using an In Vivo Image System (IVIS spectrum in vivo imaging system, PerkinElmer; Hopkinton, MA, USA) at 7, 14 or 21 days. Tumor-bearing mice were sacrificed when the animals displayed any symptoms of dragging legs, decreased activity and tumors were collected. Mouse brains were harvested and examined by IHC.

For in vivo siRNA treatment U87-MG cells (1×10^ 6^) suspended in 100 μl of PBS were subcutaneously injected into the dorsal surfaces of 5-week-old nude mice that had been previously anesthetized. Ten days after implantation, tumors were treated with siNC or siTRIM21 together with atelocollagen (AteloGene, KOKEN, Tokyo, Japan) to facilitate siRNA introduction into tumors. Briefly, A 200 μl volume of siRNA solution (30 μmol/L in 0.5% (v/v) atelocollagen) was injected directly the base of each tumor. The animal experiments were approved by the Ethics Committee of Third Military Medical University.

### Statistical analysis

All experiments were performed in at least three independent replicates and reported as the mean ± the standard error of the mean. The statistical significance was calculated utilizing an unpaired two-tailed Student's t test for direct comparisons and ANOVA for multi-group comparisons. Survival curves were compared between groups using the log-rank test. As indicated in the figure legend, all significant statistical differences were defined as *p < 0.05; **p < 0.01; ***p < 0.001.

## Results

### High level of TRIM21 expression in glioma is associated with poor prognosis

**Figure 1:**
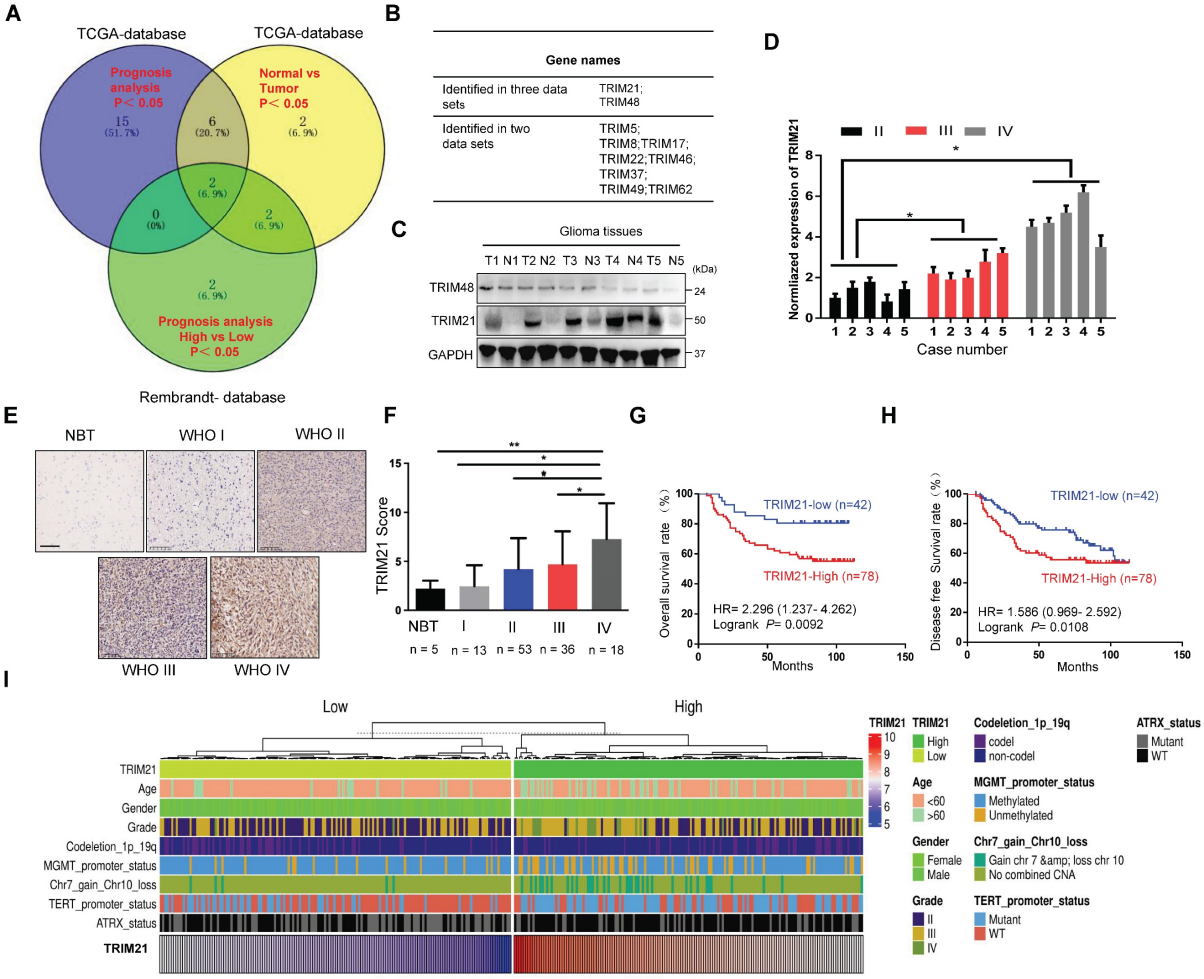
** TRIM21 is highly expressed in GBM samples and correlates with poor prognosis. (A)** Venn diagram showing the number of changed proteins from TRIMs family identified in TCGA-datasets in GBM relative to normal brain tissue samples (Blue) and the prognosis difference using Kaplan-Meier curves in GBM from TCGA-database (Yellow) and Rembrandt-Database (Green) and overlapping proteins in the datasets. **(B)** TRIM21 and TRIM48 were identified.** (C)** Western blotting of TRIM21 and TRIM48 protein in 5 paired surgically removed glioma (T) and the corresponding adjacent normal tissues (N). **(D)** The mRNA of TRIM21 in glioma in different grades. **(E-F)** Representative IHC staining images **(E)** and score **(F)** of TRIM21 in WHO grade I-IV glioma tissue microarrays and normal brain tissues (NBT). Scale bar, 100 μm. **(G-H)** Kaplan-Meier curves showing the relationship between the expression of TRIM21 and the overall survival rate **(G)** and Disease-free survival rate **(H)** of patients with glioma (n = 120). **(I)** Heatmap showing the distribution of clinical features and genetic characteristics and TRIM21 expressions in glioma specimens from TCGA-LGGGBM database (n = 330).

To identify potential oncogenes or tumor suppressor genes in the TRIM family, we analyzed the expression and prognostic value of about 80 TRIM members using the TCGA-Database and Rembrandt-Database and the following two criteria: Firstly, the expression level of the gene(s) was significantly up-regulated or down-regulated in GBM compared to normal brain samples. Secondly, the particular TRIM expression change was significantly correlated with overall survival (OS) of GBM patients. Based on these guidelines, TRIM21 and TRIM48 were identified as candidate cancer-relevant TRIM genes from our database screen (Figure 1A-B and Figure S1A-B). To further validate the bioinformatics analysis, TRIM21 expression was confirmed to be more highly expressed in glioma tumor tissues than in corresponding para-cancerous tissues by WB, whereas TRIM48 western blot experiments did not replicate the datamining observations (Figure 1C and Figure S1C). As such, among the TRIM family members, TRIM21 was determined to be an important candidate oncogene in GBM.

To further explore the correlation between TRIM21 expression and pathological characteristics, we evaluated the association of TRIM21 with glioma classification and found its mRNA was positively related with glioma grade (Figure 1D). IHC staining on a biospecimen collection that included 120 glioma samples and 5 normal brain tissues was also performed. As anticipated, TRIM21 markedly increased in Grade IV GBM material in comparison with Grade III **(**P < 0.05), Grade II **(**P < 0.05), Grade I **(**P < 0.01) or Normal brain samples (P < 0.05; Figure 1E-F and Table 1), which was also observed in TCGA database (Figure S1D). Additionally, high TRIM21 expression predicted significantly reduced overall survival (OS) and disease-free survival (DFS) rates in patients with glioma (Figure 1G-H). Multivariate analyses revealed that TRIM21 is an independent indicator for OS of patients with glioma (Figure S1E), Relative to gliomas in the TRIM21 Low group, gliomas with high expression of the TRIM21 exhibited more GBM and Chr.7.gain/Chr.10.loss, fewer IDH mutations, ATRX mutation, MGMT methylation, and 1p/19q co-deletion (Figure 1I and Table 1). Taken together, abundance TRIM21 may contribute to progression of glioma in cancer-affected individuals.

### RING E3 ligase activity is required for TRIM21-mediated GBM cell progression

**Figure 2:**
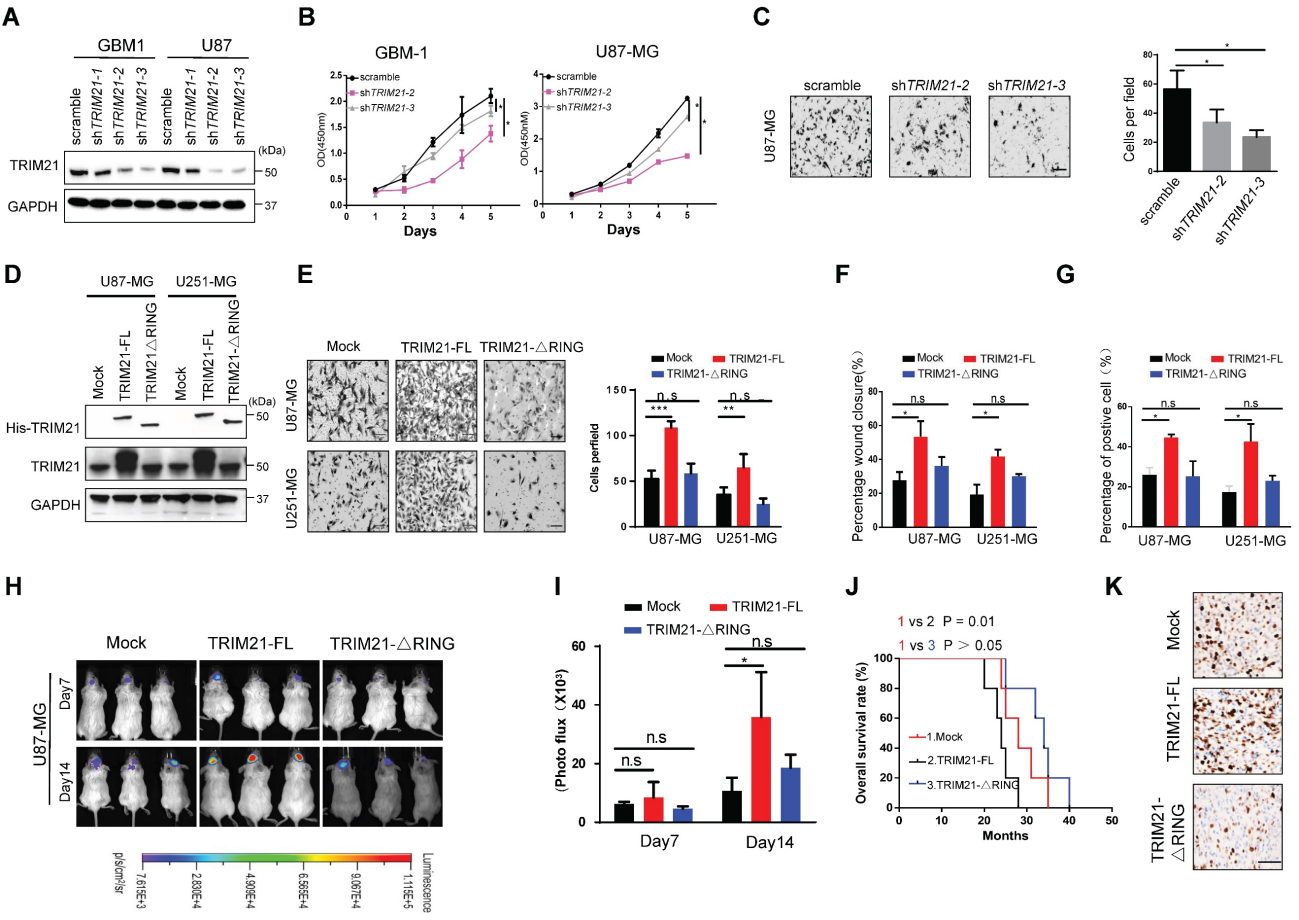
** TRIM21 facilitated invasion and growth of GBM via its RING domain in* vivo* and in *vitro*. (A)** Expression of TRIM21 protein in GBM cells (GBM-1 and U87-MG) transfected with Scramble or sh*TRIM21*. **(B)** The growth curve of GBM cells GBM cells (GBM-1 and U87-MG) transfected with Scramble or sh*TRIM21*. **(C)** Representative images (*Left panel*) and number (*Right panel*) of U87-MG cells transfected with Scramble or sh*TRIM21*. Scar bars, 50μm. **(D)** Western blot analysis of TRIM21 in GBM cells (U87-MG and U251-MG) transfected with Mock, TRIM21-FL, TRIM21-△RING plasmid. **(E)** Representative images (*Left panel*) and number (*Right panel*) of Mock, TRIM21-FL, TRIM21-△RING GBM cells (U87-MG and U251-MG) in transwell assay. **(F-G)** Summary graph indicates the motilities **(F)** and Brdu-positive immunofluorescence staining **(G)** of GBM cells transfected with Mock, TRIM21-FL, TRIM21-△RING plasmid. **(H-I)** Bioluminescent images** (H)** and the quantification (**I**) of tumors in mice implanted with Mock-, TRIM21-FL-, or TRIM21-△RING- U87-MG cells. The bioluminescent images were obtained at day 7 and 14 after injection. **(J)** Survival curves of tumor-bearing mice implanted with indicated cells. **(K)** Representative IHC staining of the tumor tissues were performed with anti-Ki-67 antibodies. Scar bars, 100μm

To evaluate potential functions of TRIM21 in glioma, GBM cells (U87-MG and GBM-1) with TRIM21 depleted were established via lentiviral particle transfection (Figure 2A and Figure S2A). GBM cells with TRIM21 depleted exhibited a reduced proliferation rate and mobility as determined by CCK8, transwell assay, and Wound-healing assays respectively (Figure 2B-C and Figure S2B).

To determine the importance of the RING E3 ligase function of TRIM21, we constructed TRIM21 mutants, one with RING domain deletion (TRIM21-ΔRING) and one as full-length (FL) and then assessed biological consequences following transfection into U87-MG or U251-MG (Figure 2D and Figure S2C). Invasiveness, mobility and proliferation were elevated in TRIM21 overexpressing (TRIM21-FL) GBM cells compared with mock cells, but remained unchanged in TRIM21-ΔRING -GBM cells respectively (Figure 2E-G; Figure S2D-E). Additionally, we implanted U87-mock, U87-TRIM21-FL, or U87-TRIM21-ΔRING cells into the brain of NOD-SCID mice and measured tumor progression. Bioluminescence imaging indicated that larger tumors were formed with U87-TRIM21-FL cells as compared with either TRIM21-ΔRING or TRIM21-mock cells (Figure 2H-I). The survival time of tumor-bearing mice was shorter in the U87-TRIM21-FL group but remained stable in the U87-TRIM21-ΔRING group as compared to the U87-mock group **(**Figure 2J). As shown in Figure 2K, Comparable results were obtained following Ki-67 index staining. Taken together, these data indicated that TRIM21 may act as an oncogene through its RING E3 ligase activity in GBM.

### TRIM21 induces β-catenin expression and nuclear translocation by mediating its K63 polyubiquitination

**Figure 3:**
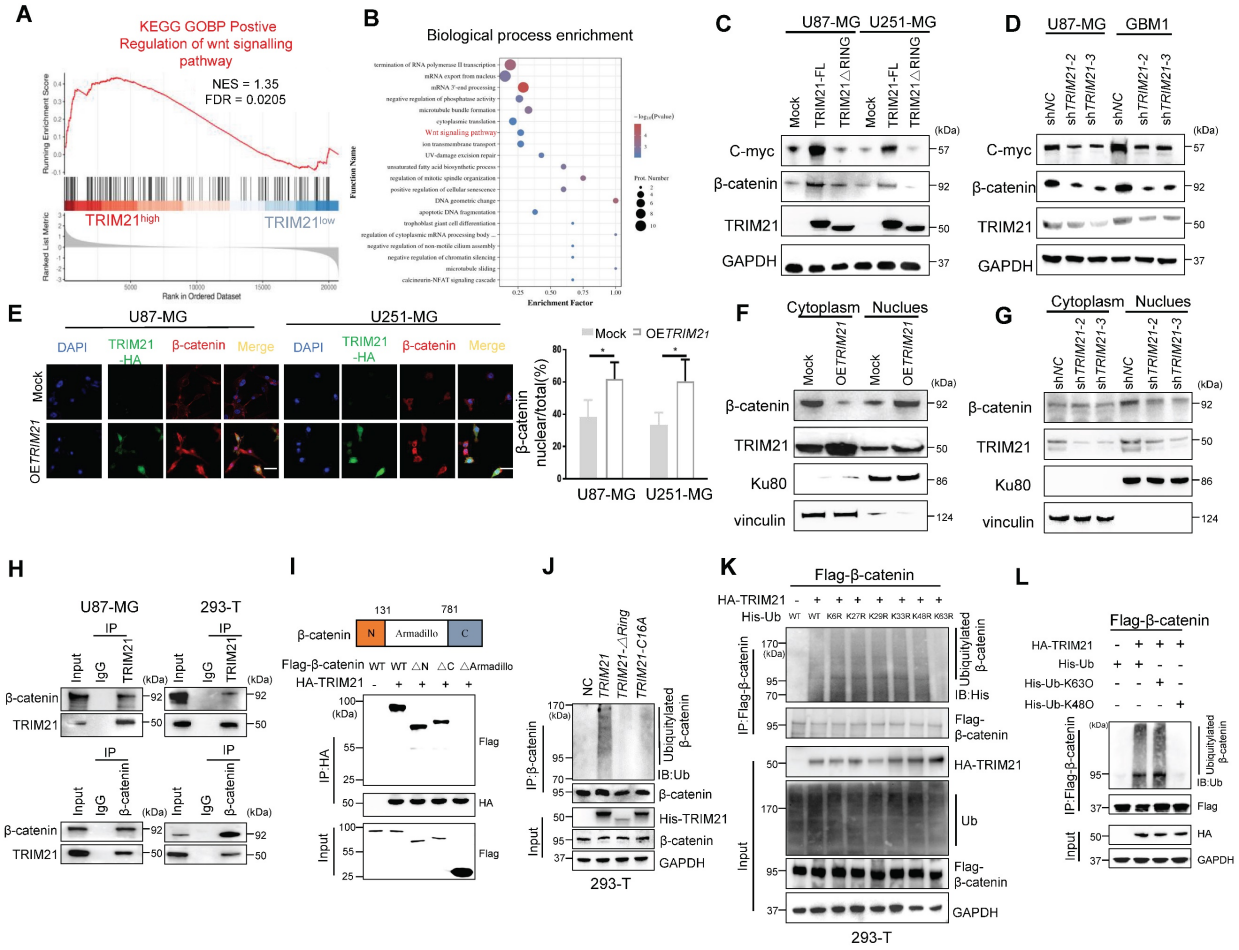
** TRIM21-mediated WNT/β-catenin through K63 polyubiquitination of β-catenin. (A)** GSEA of positive regulation of WNT signaling pathway score in human GBMs from the GSE108474 (n = 110). **(B)** Gene oncology enrichment analysis of identified Differential Expression Analysis (DEG) by TMT from U87-MG. **(C)** Western blot analysis to evaluate β-catenin and C-myc in lysates prepared from mock, TRIM21-FL and TRIM21-△RING-GBM (U87-MG and U251-MG) cells. **(D)** Western blot analysis to evaluate β-catenin and C-myc in lysates prepared from TRIM21 knock down. **(E)** Immunofluorescence (*left*) and quantitative analysis (*right*) of β-catenin nuclear translocation induced by overexpression of TRIM21. Scale bar,20 µm.** (F-G)** Overexpression **(F)** and silencing** (G)** of TRIM21 enhanced and abolished the nuclear translocation of β-catenin, respectively. β-catenin protein in the cytoplasm and nucleus were assayed in U87-MG cells with up- or down- of TRIM21 by a nuclear extraction assay. **(H)** Immunoprecipitation was performed with U87-MG-OE*TRIM21*-Flag (*Left panel*) and HEK293-T (*Right panel*) lysates using Flag or β-catenin antibodies, and the precipitants were measured by western blotting with the indicated antibodies. **(I)** The Armadillo domain of β-catenin mediates the interaction of this protein with TRIM21 (*Top panel*), Schematic illustration of β-catenin. *Bottom panel,* β-Catenin deletion mutants were co-expressed with HA-TRIM21 in 293T cells. the cells were subjected to IP with a HA antibody followed by immunoblotting (IB) with FLAG and HA antibodies. **(J)** *In vivo* ubiquitination assays performed in HEK 293T cells with mock, TRIM21, TRIM21-△RING plasmid, TRIM21-C16A, which then were transfected as indicated. Cells were treated with MG132 (10 mM for 4 h) and prior to lysis and then subjected to anti-β-catenin IP followed by anti-UB with immunoblot analysis.** (K)** His- Ub (WT, K6R, K27R, K29R, K33R, K48R, K63R) mutants were expressed with or without HA-TRIM21 in HEK293T cells. **(L)** HEK293T cells were transfected with Flag-β-catenin and HA-TRIM21 together with wild-type (WT) ubiquitin with K48O and K63O respectively. Cells were treated with MG132 (10 mM for 4 h) and prior to lysis and then subjected to anti-β-catenin IP followed by anti-His with immunoblot analysis.

With gene set enrichment analysis (GSEA) conducted on GEO database, genes associated with positive regulation of the WNT signaling pathway were enriched in TRIM21-high tissue samples relative to TRIM21-low tissue samples (Figure 3A). As shown by GO analysis our TMT results from mock and OE*TRIM21* cell (Figure 3B). Then, the level of β-catenin and c-myc, which were two Wnt//β-catenin pathway target genes, increased following overexpression of TRIM21-FL, a phenomenon not seen in GBM-TRIM21-ΔRING cells (Figure 3C and Figure S3A). Conversely, TRIM21 depletion by shRNA in GBM cells caused reduced β-catenin and C-myc protein (Figure 3D and Figure S3B). Moreover, immunofluorescence anlysis showed high TRIM21 levels associated with β-catenin distribution in the nucleus (Figure 3E). Similarly, in nuclear fractionation experiments, up- or down-regulation of TRIM21 expression in U87-MG cells resulted in elevated or decreased nuclear β-catenin respectively (Figure 3F-G and Figure S3C-3D). Thus, TRIM21 not only induces β-catenin expression but promotes its nuclear translocation.

To explore the biological significance of the TRIM21-mediated Wnt/β-catenin, the activation T-cell factor/β-catenin transcription assays was further assessed. The transcriptional activity of β-catenin was up-regulated upon overexpression of TRIM21-FL, a cellular phenotype not observed with TRIM21-ΔRING (Figure S3E). In the canonical Wnt pathway, phosphorylation of β-catenin by GSK-3β followed by binding of β-TrCP results in β-catenin degradation. Therefore, TRIM21 is likely involved in β-catenin nuclear translocation, based on which, we examined whether the TRIM21- mediation β-catenin activation involved GSK-3β. Towards that end, we used the constantly active β-catenin S33Y mutant, which is insensitive to GSK-3β-mediated phosphorylation 22, the transcriptional activity of both WT (β-catenin WT) and the S33Y mutant (β-catenin S33Y) significantly increased following up-regulated of TRIM21 (Figure S3F). Moreover, TRIM21 also significantly increased Wnt3a-induced β-catenin transactivation in U87-MG cells (Figure S3G). What mentioned above indicates that activating β-catenin induced by TRIM21 is dependent on Wnt/GSK-3β cascade.

Previous studies showed that K63 polyubiquitination of β-catenin is essential for nuclear translocation, which was also observed in YAP, NRIF, and ERK 23-25. Indeed, growing evidence indicates that β-catenin is translocated and activated by K63-linked polyubiquitination 26. Interestingly, no significantly difference in β-catenin mRNA were observed when U87 cells were knockdown or forced of TRIM21(Figure S4A). We therefore speculated that the E3 ubiquitin-protein ligase TRIM21 promotes β-catenin nuclear translocation by assembling K63 polyubiquitination chains. Immunoprecipitation assays revealed that TRIM21 exists within a common complex with β-catenin *in vivo* (Figure 3H). Actually, the expression of a series of TRIM21 truncation mutants revealed that the B-BOX2 domain is vital for TRIM21 to bind to β-catenin, while experiments with β-catenin deletion mutants showed that the Armadillo domain of β-catenin interacted with TRIM21 (Figure 3I and Figure S4B). Additionally, we found that ubiquitination of the β-catenin protein was increased following overexpression of TRIM21, an outcome that was not observed in cell with TRIM21-ΔRING or inactive mutant TRIM21-C16A (Figure 3J). An in vitro ubiquitylation further revealed that TRIM21 directly ubiquitylates β-catenin (Figure S4C). Polyubiquitination occur at six different Lys resides within Ub 27. To ascertain whether TRIM21 promotes K63 ubiquitination, we used a panel of ubiquitin mutants in which one Lys residue was replaced with Arg. Our analysis found that modification of only the K63R mutant was impaired, supporting TRIM21-mediated K63- polyubiquitination of β-catenin (Figure 3K). As shown in Figure 3L, K63 ubiquitin, but not K48 ubiquitin, supported TRIM21-mediated β-catenin modification. Moreover, K63 ubiquitination of β-catenin was markedly reduced when TRIM21 was knocked down in U87-MG cells (Figure S4D). Located in Armadillo domain, lysine 394 of β-catenin is a major K63-ubiquitination site 28. Relative to wild type, K394R-β-catenin ubiquitination was reduced, confirming K394 may be an important site in TRIM21-mediated β-catenin ubiquitination (Figure S4E) Collectively, K63 ubiquitinated β-catenin increased in OE*TRIM21* cells, in parallel with its translocation and activation.

### TRIM21 mediates the polyubiquitylation and degradation of TIF1γ at K5 site

**Figure 4:**
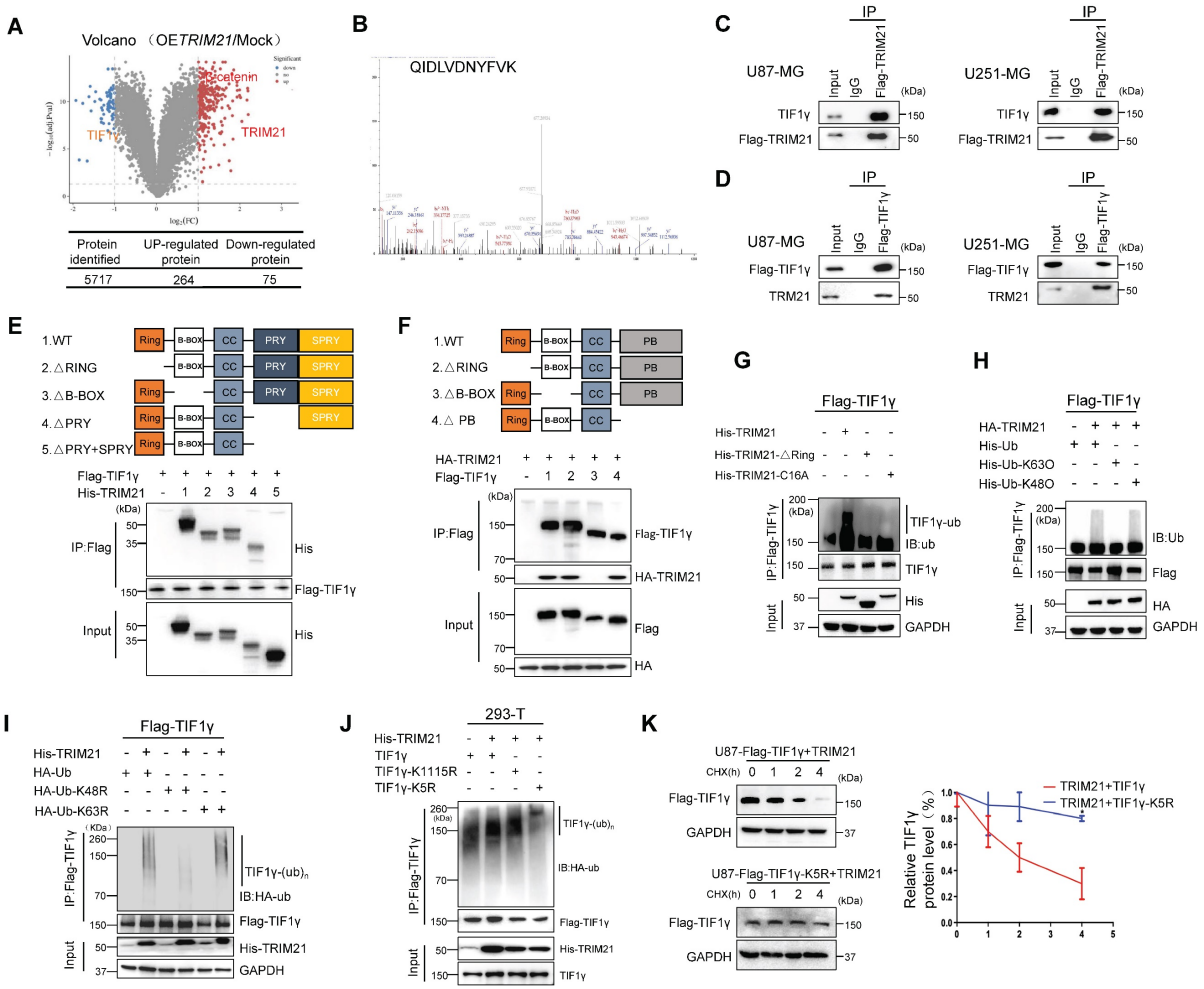
** TRIM21-mediated degradation of TIF1γ. (A)** Volcano plot of the protein abundance changes in response to OE*TRIM21*. Average protein expression ratio of 4 replicates (log 2 transformed) between TRIM21 overexpression and mock U87-MG cells was plotted against p-value by t-test (-log 10 transformed). Cutoffs of p = 0.05 and 2.0 -fold change were marked, respectively. The figure below shows the total number of proteins identified as well as the number of up- and down-regulated proteins. **(B)** MS analysis identified TIF1γ as the key interacting protein of TRIM21. TRIM21 was immunoprecipitated from U87-MG expressing HA-TRIM21 using anti-HA-conjugated beads. TIF1γ was identified through MS analysis by peptides covering the TIF1γ protein sequence. representative detected peptide (QIDLVDNYFVK) of TIF1γ was shown. **(C)** Co-IP of TRIM21 with TIF1γ in U87 cells (*left panel*) and U251-cells (*Right panel*). **(D)** Co-IP of TIF1γ with TRIM21 in U87 cells (*left panel*) and U251-cells (*Right panel*).** (E)** Full-length TRIM21 and a series of TRIM21 mutants with deletion (△) of various domains (*top panel*). 293 T cells were co-transfected with Flag-TIF1γ and WT HA-TRIM21 or their truncation mutants for 48 h. CO-IP assay was performed. **(F)** Full-length TIF1γ and a series of TIF1γ mutants with deletion (△) of various domains (*top panel*). 293 T cells were co-transfected with Flag-TIF1γ or their truncation mutants and WT HA-TRIM21 CO-IP assay was performed. **(G)** *In vivo* ubiquitination assays performed in U87-Flag-*TIF1γ* cells transfected with indicated plasmid. Cells were treated with MG132 (10 mM for 4 h) and prior to lysis and then subjected to anti-Flag IP followed by anti-UB with immunoblot analysis. **(H-I)** TRIM21 is a K48-linkage-specificubiquitin ligase for TIF1γ. HEK293T cells were transfected with Flag-TIF1γ and HA-TRIM21 together with wild-type (WT) ubiquitin **(H)**, with each of the ubiquitin mutants Ub^K48O^, Ub ^K63O^ (H), Ub ^K48R^ or Ub ^K63R^ **(I)**. **(J)** HEK293-T cells were cotransfected with the indicated plasmids for 48 h and then treated with 10 mM MG132. The polyubiquitination levels of Flag-TIF1γ and Flag-TIF1γ-K5R; Flag-TIF1γ-K1115R mutant protein were analyzed. **(K)** Left panel: U87-MG cells were transfected with TRIM21-TIF1γ-WT or TRIM21-TIF1γ-K5R plasmid for 24 h, and then treated with cycloheximide (CHX, 20 μg/mL) or the indicated times before harvesting. Cells lysates were analyzed by immunoblotting.

As noted above, the level of β-catenin increased in OE*TRIM21*-GBM cells and decreased in sh*TRIM21*-GBM cells as compared with controls (Figure 3C). Next, we identified proteins that experienced a significant change in overall level upon TRIM21 overexpression using tandem mass tag (TMT). In OE*TRIM21* cells relative to controls, 75 down regulated and 264 upregulated proteins were identified (Figure 4A). Substrates of TRIM21 might be involved in tumor progression, we carried out immunoprecipitation assays and subsequent MS analysis (IP-MS) of captured complexes (Figure 4B and Figure S4A). This analysis revealed two likely binding partners of TRIM21 and possible substrates of the E3 ligase: TIF1γ and LAMC1 (Figure S4B). SiTIF1γ or siLAMC1 were transfected into U87-MG cells, and two down-stream targets of wnt/β-catenin signaling, i.e., c-Myc and CyclinD1, were measured. Unlike in siTIF1γ knock down cells as compared to siNC cells, where c-Myc and CyclinD1 increased in the former, no difference in target protein expression was observed in siLAMC1 cells **(**Figure S4C). To this end, we identified TIF1γ, another glioma associated tumor suppressor and E3 ligase for β-catenin 14, regulates the inactivation of Wnt/β-catenin cascade, our data support that TIF1γ may act as a substrate in the TRIM21-mediated response.

Focusing on the TRIM21-TIF1γ interaction, co-immunoprecipitation assays further supported interactions between ectopic TRIM21 and TIF1γ (Figure 4C). Moreover, transfection of Flag-TIF1γ was conducted in the GBM cell lines, and immunoprecipitation experiments with anti-flag showed an interaction between ectopic TIF1γ and endogenous TRIM21 (Figure 4D). Immunofluorescence staining and CO-IP using cytoplasmic and nuclear fractions revealed that TIF1γ co-localized and interacted with TRIM21 in nuclei, whereas not in cytoplasm (Figure S4D-E). The expression of a series of TRIM21 truncation mutants with deletion (Δ) of various domains in 293T cells revealed that the C-terminal PRYSPRY domain is necessary for the binding of TRIM21 to TIF1γ (Figure 4E). Furthermore, the interaction between TRIM21 and TIF1γ was mediated by the N-terminal B-BOX domain of TIF1γ as assessed using TIF1γ deletion mutants (Figure 4F). Thus, the results indicate that the TRIM21 PRYSPRY domain interacts, possibly directly with the TIF1γ B-BOX domain.

Since both TRIM21 can promote the proliferation and migration of GBM, which could also interact with TIF1γ, we investigated a potential effect of TRIM21 on the expression of TIF1γ. Forced TRIM21 in GBM cells caused a reduction in TIF1γ protein levels (Figure S4F), but not in TIF1γ mRNA levels (Figure S4G). We therefore speculated that the regulatory effect of TRIM21 on TIF1γ protein might be through affecting its stability. Consistently, in U87 cells transfected with an expression plasmid encoding Flag-TIF1γ, TRIM21 overexpression dramatically increased the ubiquitination of TIF1γ in the presence of MG132, whereas the RING domain deletion or C16A of TRIM21 had no effect (Figure 4G). Using a ubiquitin mutant in which K48 or K63 has been replaced with Arg (K48R and K63R), K48R ubiquitin, which blocks the ubiquitin linkage for proteasome degradation, was unable to support TIF1γ ubiquitation, whereas K63R ubiquitin did not (Figure 4H). Complementarily, we used K48O and K63O in which one Lys residue was retained and the rest were replaced with Arg residue, and confirmed K63 ubiquitin mediated TRIM21-induced TIF1γ ubiquitation. To identify the ubiquitylation residue of TIF1γ, we analyzed the TIF1γ sequence using the webtool UbPred: predictor of protein ubiquitylation sites (http://bdmpub.biocuckoo.org/prediction.php) and found that the K5 and K1115 may have been ubiquitylated (Table S2). The TIF1γ K5R not K1115R mutant showed complete resistance to TRIM21-mediated ubiquitylation (Figure 4I), with a much longer half-life than that of its WT counterpart (Figure 4J).

### TRIM21 / TIF1γ axis is involved in β-catenin and tumor progression

**Figure 5:**
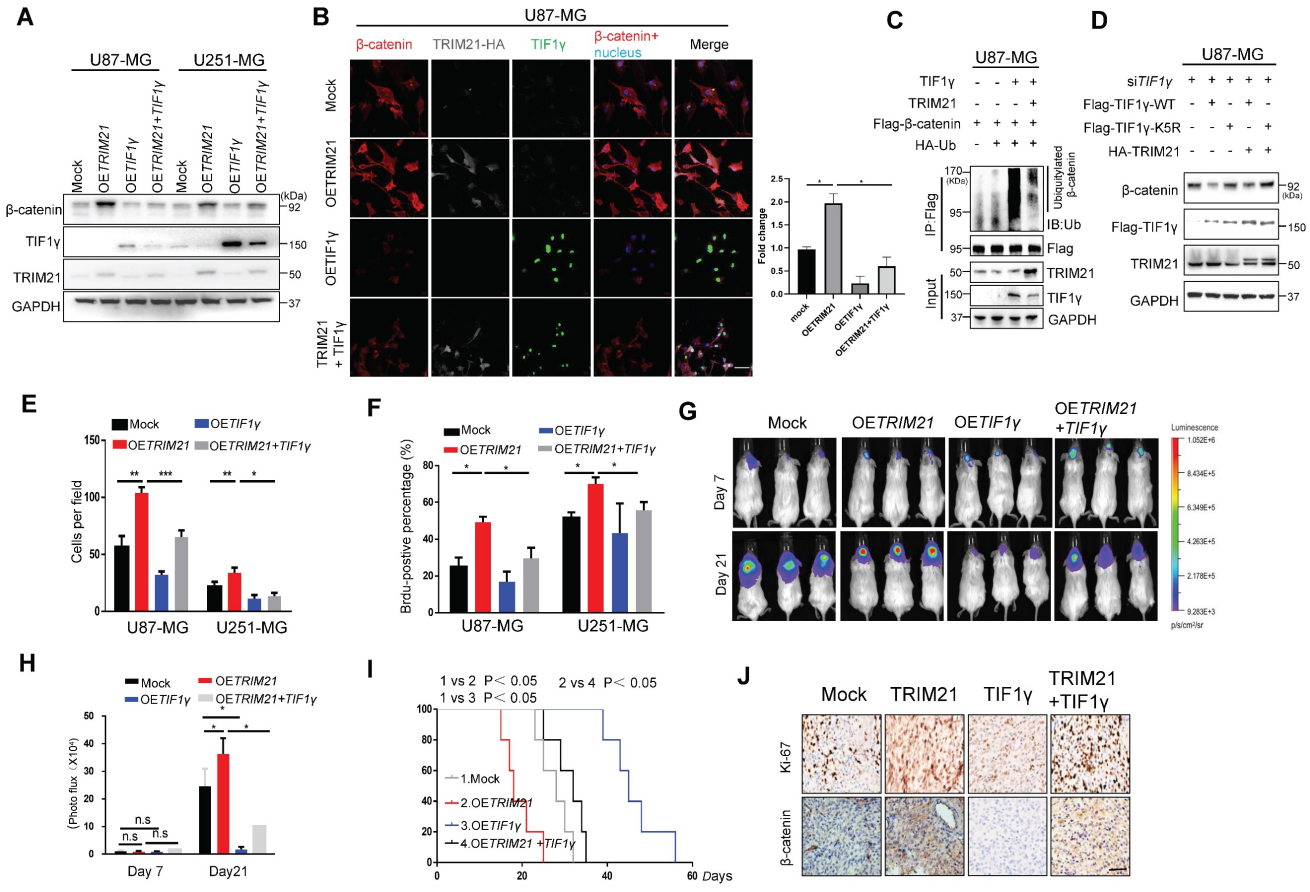
** TRIM21 / TIF1γ axis is involved in β-catenin stability and tumor progression. (A)** Immunoblotting of TRIM21, TIF1γ and β-catenin in GBM cells (U87-MG and U251-MG) overexpressing TRIM21, TIF1γ and TRIM21 + TIF1γ.** (B)** Immunofluorescence scanning of β-catenin (Red), TIF1γ(Green), TRIM21(White) in GBM cells overexpressing TRIM21 and/or TIF1γ; Scar bars, 50μm. **(C)** In vivo ubiquitination assays performed in U87-MG cells transfected with indicated plasmid. Cells were treated with MG132 (10 mM for 4 h) and prior to lysis and then subjected to anti-β-catenin IP followed by anti-UB with immunoblot analysis. **(D)** Immunoblotting of TRIM21, TIF1γ in U87-MG-siTIF1γ cells Overexpressing TIF1γ, TI*F1*γ-K5R, TRIM21+ TIF1γ, TIF1γ-K5R+ TRIM21. **(E)** The invasive number of Mock, OE*TRIM21*, OE*TIF1γ* and OE*TRIM21+TIF1γ* GBM Cells (U87-MG and U251-MG). **(F)** The number of Brdu-positive immunofluorescence staining for the indicated GBM cells.** (G-H)** Bioluminescence **(G)** and Quantification **(H)** of tumors in NOD-SCID mice implanted with Mock, TRIM21, TIF1γ and TIF1γ+ TRIM21-U87-MG cells. **(I)** Survival curves of tumor-bearing mice implanted with Mock, OE*TRIM21*, OE*TIF1γ* and OE*TIF1γ+TRIM21* U87-MG cells. **(J)** Representative IHC image of the tumor tissues were performed with anti-β-catenin and anti-Ki-67 antibodies. Scar bars, 50μm.

Considering that the β-catenin protein is a common target of TRIM21 and TIF1γ 14, we sought to determine whether this juxtaposed regulatory mechanism might contribute to the progression of GBM. Towards that end, GBM cells (U87-MG and U251-MG) were transfected with OE*TRIM21*, OE*TIF1γ*, or OE*TRIM21 + TIF1γ*. As expected based on the earlier results, upregulation of TRIM21 increased β-catenin protein levels, which were conversely significantly decreased by OE*TIF1γ* overexpression (Figure 5A-B). By contrast, decreased TRIM21 down-regulated β-catenin was observed in TIF1γ-WT cell, whereas not in TIF1γ-KD cell (Figure S6B). Moreover, β-catenin ubiquitination was elevated following overexpression of TIF1γ, which was reversed by TRIM21 overexpression in U87-MG cells (Figure 5C). Similarly, in vitro pull-down assays showed TRIM21 effectively blocked the β-catenin-TIF1γ interaction (Figure S6C). We also found TIF1γ but not TIF1γ-K5R decreased the TRIM21-mediated β-catenin augmentations **(**Figure 5D and Figure S6D). These results suggested TRIM21 degraded TIF1γ at k5 site, subsequently inhibiting β-catenin stability.

Addressing specifically the relationship of TRIM21 and TIF1γ on the progression of GBM, we highlight that TIF1γ expression decreased GBM cell mobility and growth, partially overcoming the oncogenic effects of TRIM21 on tumor invasion (Figure 5E and Figure S6E) and proliferation (Figure 5F and Figure S6F).Additionally, bioluminescence analyses revealed that TIF1γ overexpression partially reduced the stimulatory effects of TRIM21 overexpression on GBM growth in vivo (Figure 5G-H).Whereas OS was reduced in mice bearing U87-OE*TRIM21* tumors relative to controls, TIF1γ overexpression normalized OS of U87-OE*TRIM21* animals (Figure 5I). IHC staining of tumor xenografts showed that TRIM21 overexpression markedly increased β-catenin expression, which was reduced by TIF1γ overexpression, which was also observed in Ki-67 index respectively (Figure 5J and Figure S6G). These data further confirm that TRIM21 works antagonistically against TIF1γ to control β-catenin stability and thereby determine GBM progression phenotype.

### The R443W mutation eliminates the effect of TRIM21 on the expression of TIF1γ/β-catenin axis

**Figure 6:**
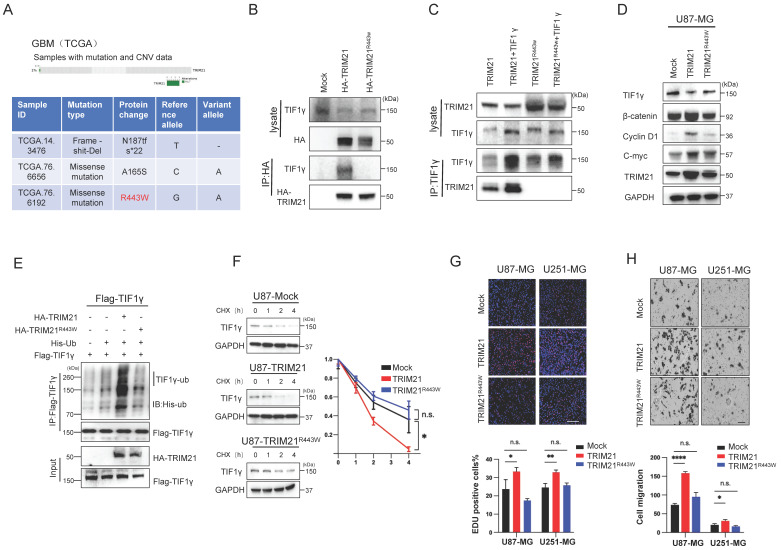
**Arginine 443 in TRIM21 is critical for mediating the ubiquitination and degradation of TIF1γ. (A)** The R443W mutation of TRIM21 was identified from TCGA GBM database (http://www.cbioportal.org). **(B)** U87-MG cells were transfected with HA-tagged TRIM21 and its mutants R443W (HA-TRIM21, HA-TRIM21^R443W^ respectively) Cell lysates were immunoprecipitated with anti-HA antibodies.** (C)** U87-MG cells were transfected with HA-tagged TRIM21, its mutants HA-TRIM21-R443W and/or Flag-TIF1γ respectively, cells were immunoprecipitated with anti-TIF1γ. **(D)** Western blot analysis to evaluate TIF1γ, β-catenin, C-myc and cyclin D1 in lysates prepared from Mock, TRIM21 and TRIM21-R443W-GBM (U87-MG) cells. **(E)** *In vivo* ubiquitination assays performed in U87-MG transfected with mock; HA-tagged TRIM21, its mutants HA-TRIM21-R443W. Cells were treated with MG132 (10 mM for 4 h) and prior to lysis and then subjected to anti-Flag IP followed by anti-HIS with immunoblot analysis. **(F)** Immunoblotting (*Left panel*) and Quantitation (*Right panel*) of TIF1γ in GBM cell (Mock, TRIM21, TRIM21^R443W^) treated with CHX (100 mg/ml) and MG132 for different times. **(G)** Brdu-positive immunofluorescence staining images (*Upper panel*) and number (*down panel*) of GBM cells transfected with (-Mock, -TRIM21, -TRIM21^R443W)^ Scar bars, 100μm. **(H)** Representative images (*Upper panel*) and number (*down panel*) of invaded U87-MG cells and U251-MG transfected with (-Mock, -TRIM21, -TRIM21^R443W^) Scar bars, 100μm.

Considering the deletion of PRYSPRY domain abolished the interaction between TRIM21 and TIF1γ, moreover, we analyzed the GBM Dataset from TCGA, and identified 3 glioma cancer samples with genomic alteration on TRIM21, including 2 missense mutations, 1 deep deletions (Figure 6A). Among the 2 samples with TRIM21 missense mutations, one had a mutation in the PRYSPRY domain of TRIM21, which caused an Arginine to Tryptophan change at position 443 (R443W), and the other had mutations on the B-BOX. As such, R443W mutation may be involved in regulating TIF1γ/β-catenin cascade. Furthermore, we transfected the HA-TRIM21 and HA-TRIM21^R443W^ constructs into U87-MG cells, and immunoprecipitation (IP) results showed that the R443W mutation abolished the interaction between TRIM21 and TIF1γ (Figure 6B). In a reciprocal assay, IP of TIF1γ pulled down the wild type, but not the R443W mutated TRIM21 (Figure 6C). While transfection of the wild type TRIM21 decreased TIF1γ and subsequently induced β-catenin expression, whereas transfection of the R443W mutated TRIM21 did not (Figure 6D and Figure S7A).

Next, we performed whether the R443W mutation could affect the TRIM21-mediated ubiquitination and degradation of TIF1γ. Transfection of the R443W mutated TRIM21 decreased the ubiquitination of TIF1γ as compared with wild type TRIM21 (Figure 6E). Consistently, transfection of wild type TRIM21 changed the turnover time of TIF1γ, but not observed in R443W mutated TRIM21, indicating that the R443W mutated TRIM21 did not affect the stability of TIF1γ protein (Figure 6F).

To investigate the underlying the functional effects of the R443W mutation on invasion and growth, we used Brdu assay and transwell assays to examine the growth speed and invasion capabilities of the GBM cells. Cells transected with the wild type TRIM21 displayed increased invasiveness and growth than Mock cells, whereas transfection of the R443W mutated TRIM21 did not (Figure 6G-H).

### TRIM21 expression correlates with β-catenin and TIF1γ expression in GBM

**Figure 7:**
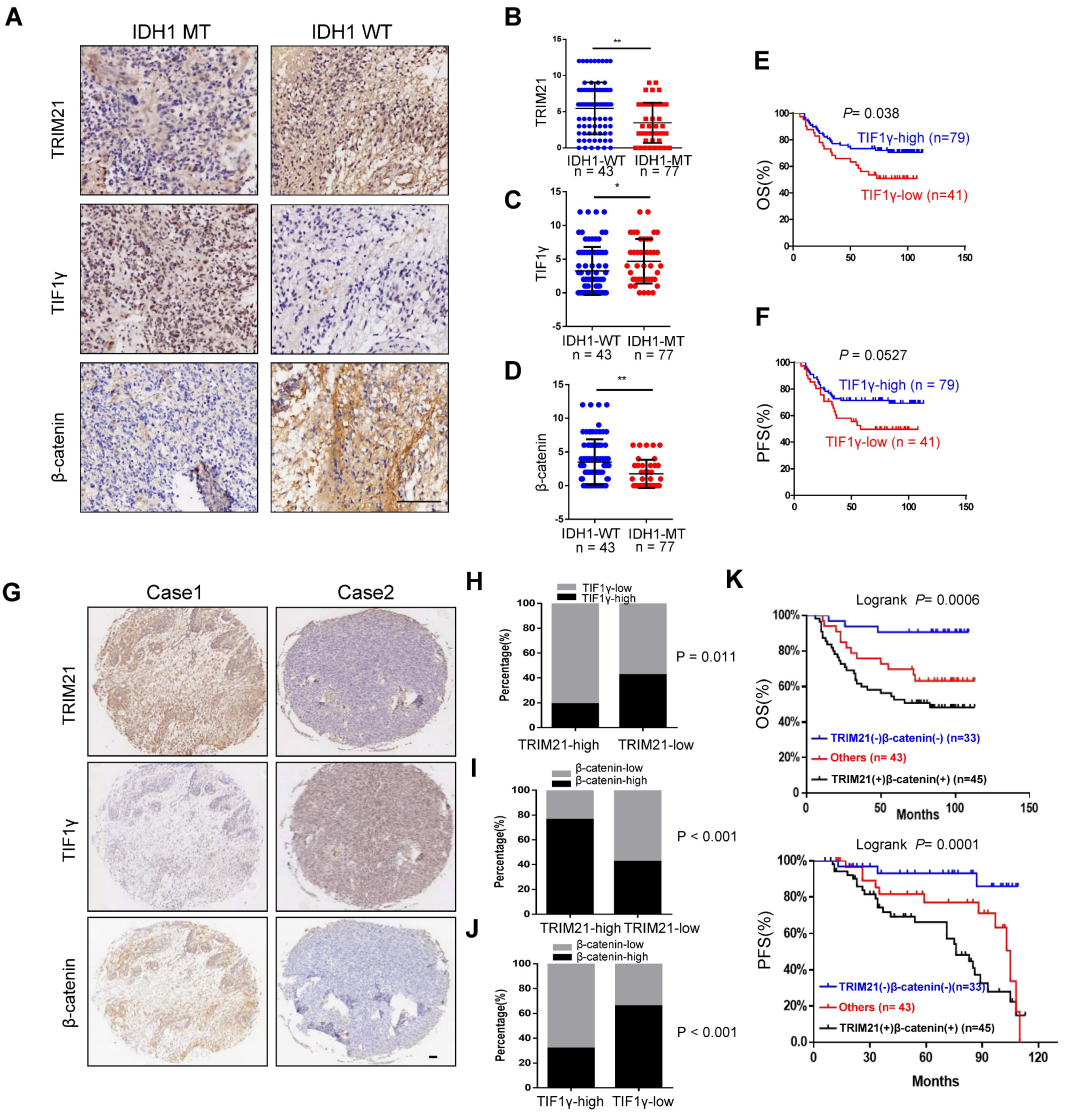
** Correlation of TRIM21, TIF1γ and β-catenin in Glioma. (A-D)** Representative image of TRIM21, TIF1γ and β-catenin expression in IDH1-WT and IDH1-MT glioma examined by IHC. Scare bars, 100 μm. **(E-F)** Kaplan-Meier plots of the OS **(E)** rates and DFS **(F)** rates in human glioma specimens in the groups with high and low expression of TIF1γ. **(G)** IHC staining of 120 human glioma specimens was performed with the indicated antibodies. Representative images from the staining of TRIM21, TIF1γ and β-catenin were shown scar bars, 100μm. **(H)** χ^2^ analysis showing the percentage of TIF1γ-high and TIF1γ-low in TRIM21-high group and TRIM21-low group. **(I)** χ2 analysis showing the percentage of β-catenin-high and β-catenin-low in TRIM21-high group and TRIM21-low group. **(J)** χ2 analysis showing the percentage of β-catenin-high and β-catenin-low in TIF1γ-high group and TIF1γ-low group. **(K)** Kaplan-Meier plots of the overall survival rates and Disease-free survival rates in human glioma specimens in the groups with TRIM21(+) β‐catenin (+), TRIM21(-) β‐catenin (-), and others.

To evaluate the clinical importance of the TRIM21/TIF1γ/β-catenin axis and determine their correlation in glioma, we analyzed 120 human glioma specimens with using IHC staining. Furthermore, IDH1 mutation was an important classification biomarker for glioma 29, The IHC results showed that compared with the IDH1-MT, the expression levels of TRIM21 and β-catenin are significantly upregulated but TIF1γ decreased in IDH1-WT glioma (Figure 7A-D). In particular, patients bearing brain tumors with relatively low levels of TIF1γ and high level of β-catenin showed shorter overall survival and progression survival than those with others respectively (Figure 7E-F). Furthermore, TRIM21 protein levels were positively correlated with β-catenin expression, in addition, TRIM21 and β-catenin expression levels were inversely correlated with TIF1γ protein levels respectively (Figure 7G-7J). Interestingly, Kaplan-Meier survival analysis of cohort clinical GBM specimens suggested that the patients with TRIM21^-high^ and β-catenin^-high^ showed shorter overall survival (OS) and progression-free survival (PFS) as compared with others and TRIM21-low and β-catenin-low group (Figure 7K). Thus, our data indicate that TRIM21 in combination with β-catenin could serve as a valuable prognostic biomarker set for glioma patients. Importantly, our data also indicate that the TRIM21/TIF1γ/β-catenin axis plays a critical role in the clinical behavior of human glioma.

### Inhibition of xenograft tumor proliferation by in vivo treatment with TRIM21 siRNA

**Figure 8:**
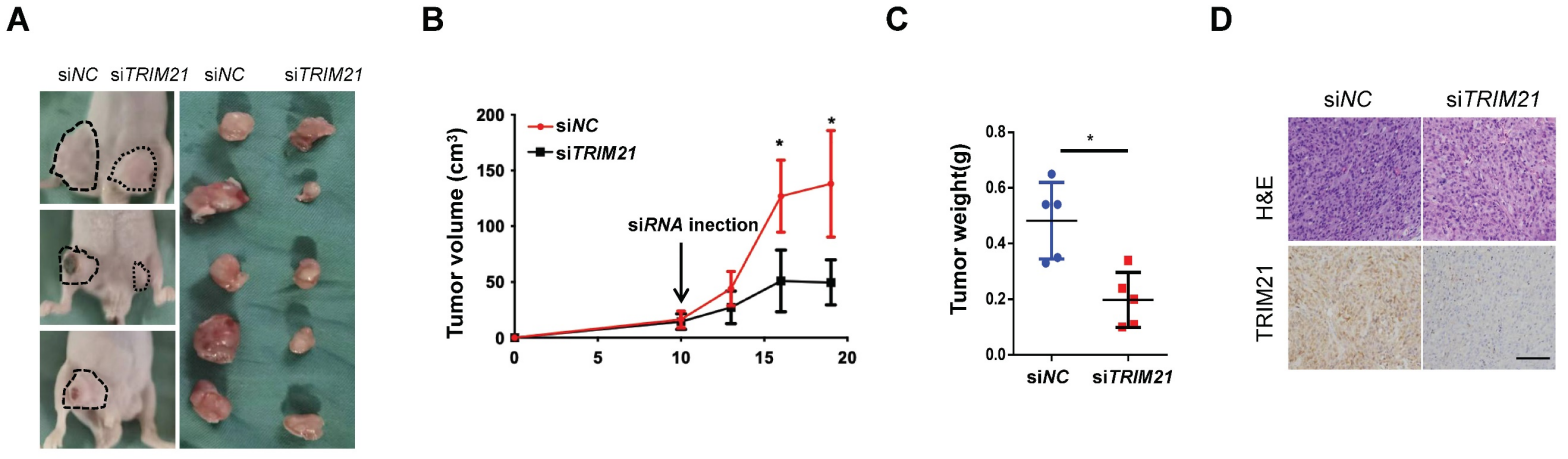
** Inhibition of xenograft tumor proliferation by in vivo treatment with siRNA. (A-C)** U87-MG cells (1 × 10^6^ cells) were subcutaneously implanted into nude mice. After 10 days, control or TRIM21 siRNA was injected into the developed xenograft tumors (n = 5). 10 days after siRNA injection, the nude mice were killed. Dashed lines show the outline of xenograft tumors in representative three mice (*Left picture*), and extirpated xenograft tumors are shown (*Right pictur*e) **(A)**. The volumes **(B)** and weights **(C)** of five pair of tumors were measured. **(D)** Sections were stained with TRIM21 Scar bars, 50 μm.

We examined TRIM21 as a potential target for cancer therapy. U87-MG cells were implanted into nude mice, and tumors were allowed to develop for 10 days (tumor diameter is ~ 8 mm). Next, control or TRIM21 siRNA was mixed with atelocollagen and injected in tumor every day. After ten days, the nude mice were sacrificed. Such in vivo treatment with TRIM21 siRNA reduced the volumes and weights of the tumors (Figure 8A-C) and also clearly decreased the TRIM21 in xenografts compared with levels in tumors injected with control siRNA (Figure 8D). Therefore in vivo treatment with TRIM21 siRNA reduced tumor sizes, indicating TRIM21 as a therapeutic target for glioma.

## Discussion

IDH1-WT GBM, accounting for almost 90% of all GBM, features with high WNT/β-catenin pathway activity 29. Disrupting β-catenin stability has been a major strategy in inactivating WNT signaling to prevent the malignant behavior of GBM 30, 31. Notably, a prior clinical study demonstrated that β-catenin inhibitors initiate a broad range of responses that are effective at treating acute myeloid leukemia. Thus, targeting Wnt/β-catenin has emerged as a promising therapeutic method for cancer, including GBM. In our research, up-regulated in IDH1-WT gliomas, TRIM21 was associated with advanced tumor grade and poor prognosis. We highlighted that TRIM21 is needed for GBM progression and poor prognosis as well as aids in the generation of K63-linked Ubiquitin conjugates of β-catenin, a modification that in turn promotes β-catenin translocation to the nucleus. TRIM21 also promoted K48-linked ubiquitination of TIF1γ, a key negative regulator of Wnt/β-catenin signaling, supporting reduced β-catenin ubiquitination and increased its levels (Figure 8E). However, TRIM21 degrades β-catenin in liver cancer 32, and switching of polyubiquitination of β-catenin from K48 to K63 were also observed in CRC 33. Taken with our study, these findings indicated the style of β-catenin ubiquitination was striking discrepancy in different tumor.

TRIM21, an autoantigen that presents in patients with systemic lupus erythematosus (SLE), encodes for a ubiquitously expressed protein known as RING dependent E3 ligase that can be detected in the cell cytoplasm and nucleus 34. It also promoted 35 or impeded 36, 37 cancer progression via ubiquitination of target cancer-related proteins and releasing DNA mitochondrial 6 TRIM21 was also reported to promote invasion and radiotherapy resistance in glioma 38, 39. Herein, we determined that TRIM21 facilitated the progression of GBM in vitro and in vivo via its RING-dependent E3 ligase activity, and that PRYSPRY domain of TRIM21, was critical for the interaction with TIF1γ and its degradation. To our knowledge, PRY-SPRY domain is a requisite for TRIM21-mediated cancer progression 40, 41. Specifically, the PRY-SPRY domain of TRIM21 binds to the Fc motif of antibodies and triggered the acute degradation of endogenous proteins without modification of the genome or mRNA via a so-called TRIM-away system 42. Notably, the TRIM-away system has been applied to a wide range of target proteins using off-the-shelf reagents. Additionally, we identified a novel mutant, TRIM21R443W form, and demonstrated that the R443W mutation from PRY-SPRY domain abolishes the TRIM21-mediated progression in GBM via TIF1γ/β-catenin axis. Consistent with our finding, R64Q mutation also decreased TRIM21-mediated invasion in breast cancer 40. These results suggested mutations of TRIM21 were associated with tumor progression. As highlighted by our work, TRIM21 is a promising therapeutic target in GBM owing to its unique structure and operational functions.

Studies indicated that TRIM21 ubiquitinates both itself and TRIM5a through its ligase activity, yet does not facilitate proteasomal degradation 43. We demonstrate that TRIM21 ubiquitinates TIF1γ at K5 site, another TRIM family member, leading to proteasomal degradation, indicating that TRIM proteins form a complex network of distinct intra- and inter-regulation. The family of TRIM proteins have been classified into six types based on their diverse C-terminal domains 10. For instance, TRIM5a and TRIM21, which share high structural and functional similarities, are prototypes of the evolutionarily conserved class IV category 43, whereas TIF1γ belongs to class V TRIM proteins which possess PHD and Bromo C-terminal domains. Thus, the manner in which TRIM21 and other family members mediate ubiquitination of themselves or the other TRIM proteins is seemingly correlated with the molecular composition of their substrates. Additionally, the Ad5 E4-ORF3 protein can promote the initial conjugation of SUMO3 to TIF1γ, inducing its sumoylation and proteasomal degradation 44. Together with our findings, TIF1γ stability was of importance in tumor progression.

The β-catenin pathway is one of the most commonly activated and mutated in GBM. TRIM21 is essential for tumor growth and invasion of GBM cells and its upregulation may correlate with β-catenin activation. These results indicate a potential use of TRIM21 as a drug target and a prognostic marker for GBM driven by β-catenin hyper-activation.

## Supplementary Material

Supplementary figures and tables.

## Figures and Tables

**Table 1:** The correlation between TRIM21 expression and clinicopathological factors of patients with glioma.

Feature		TRIM21 (Low)	TRIM21(High)	
		N=42	N=78	*P* value
Gender				
Male		29	48	*P* = 0.413
Female		13	30	
Age				
≥ 50		23	34	*P* = 0.242
< 40		19	44	
Prepredominant lobe of tumor location				
Frontal		10	23	
Temporal Parietal Others		17	25	
		2	7	*P* = 0.673
		13	23	
Grade				
I+II III+IV		29	38	
		13	40	***P* = 0.032**
IDH1 status WT MT		21 21	56 22	***P=*0.027**
Ki67				
< 20%		33	67	*P*=0.304
>= 20%		9	11	

NOTE: The bold values are statistically significant (*P* < 0.05)
