# Supplementary material for: E3 ubiquitin ligase TRIM21 targets TIF1γ to regulate β-catenin signaling in glioblastoma
Source: Theranostics. 2023 Sep 4;13(14):4919–35. doi: 10.7150/thno.85662 (PMC10526654; doi:10.7150/thno.85662)
Supplement: Supplementary file 1 — Supplementary figures and tables. [file thnov13p4919s1.pdf]

Supplementary Material

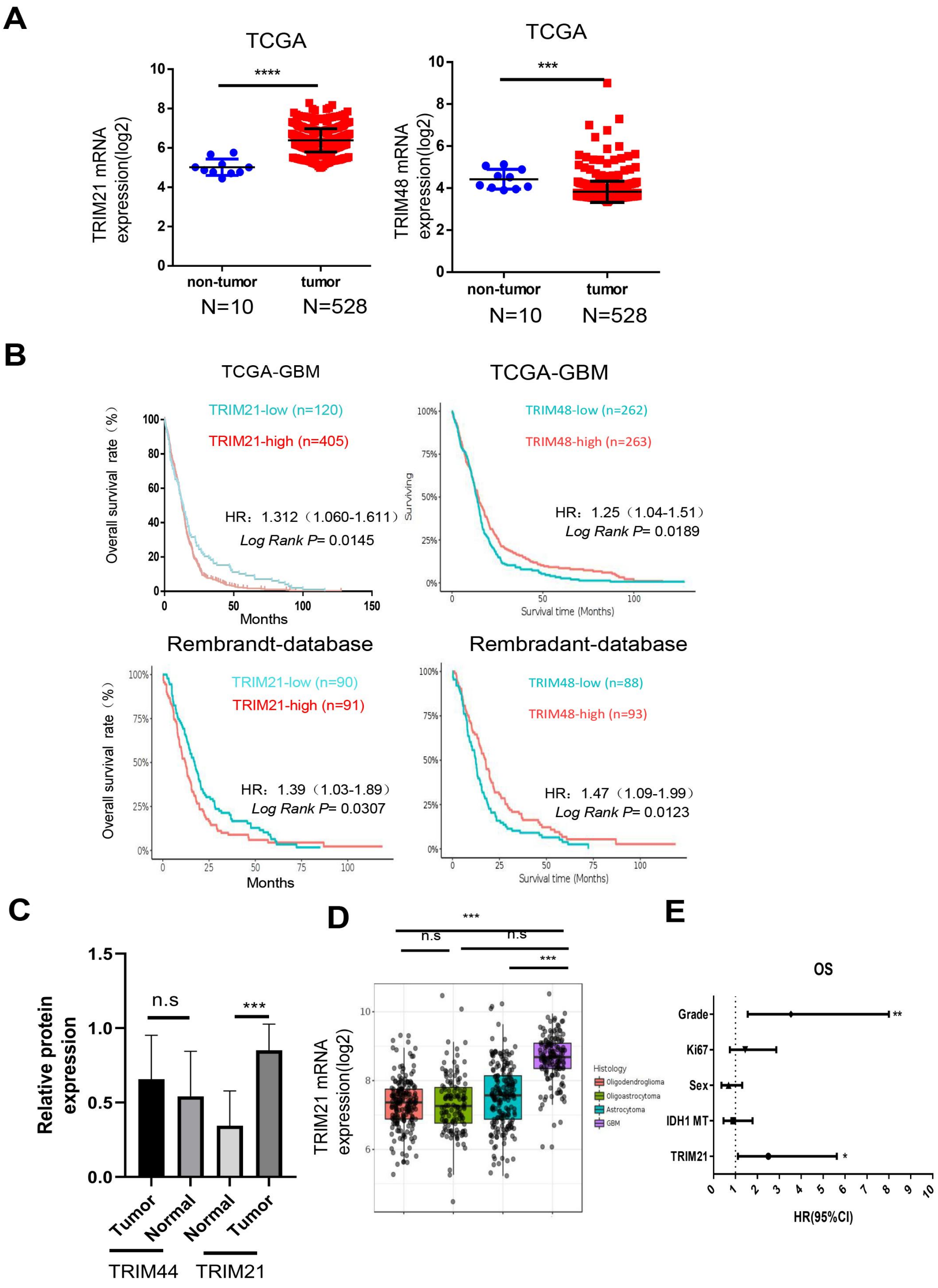

Supplementary Figure 1： The expression and prognostic of TRIM21 and TRIM48 in glioma.

(A) The expression of TRIM21 and TRIM48 in NBT and GBM in

TCGA-Database. (**B)** Kaplan-Meier curves of the overall survival rate of GBM patients with high versus low TRIM21 (*Left*) /TRIM48 (*Right*) expressing tumors in TCGA-Database （*Left panel*） and Rembrandt-Database (*Right panel).* (**C)** The qualification analysis show TRIM21 and TRIM48 expression levels in 5 paired glioma tissue. (**D)** The TRIM21 mRNA in different-grade glioma tissue from TCGA-database. **(E)** Multivariate analysis for OS in glioma patients.Data are presented as means ± SEM. **p $<$ 0.01, ***p$<$0.001.

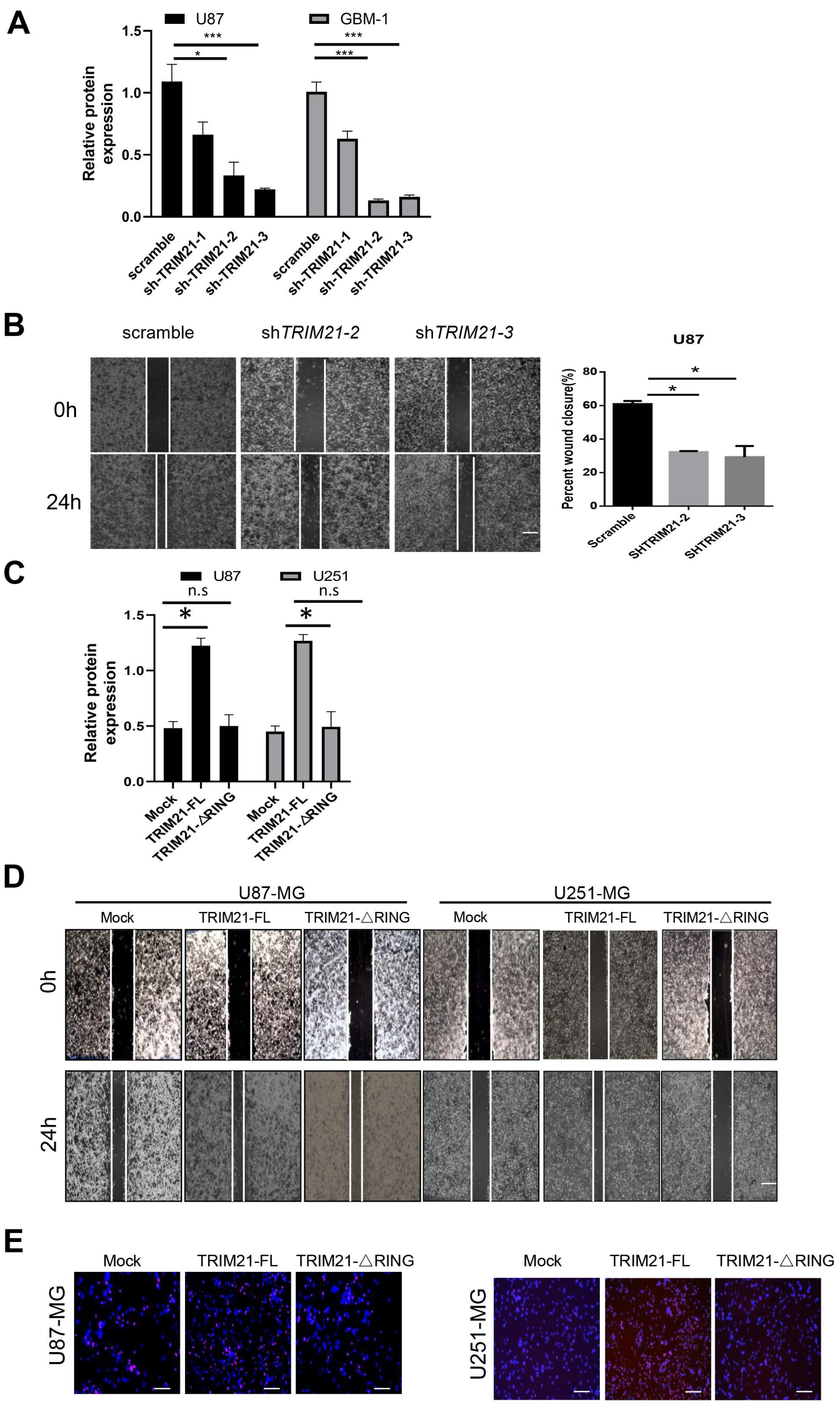

Supplementary Figure 2: TRIM21 is critical for tumor progression *in*

vitro

(A) Quantitation of TRIM21 in U87-MG transfected with vector or shTRIM21 lentivirus. (**B)** Images (*Left panel*) and summary graph (*Right panel*) indicated the motilities of U87-MG cells transfected with scramble, sh*TRIM21-2*, sh*TRIM21-3* by Wound healing experiments. Scar bars, 100μm. (**C)** Quantitation of TRIM21 in U87-MG transfected with mock, TRIM21 and TRIM21-△RING plasmid respectively. Scar bars, 100μm (**D)** Representative images indicated the motilities of GBM cells transfected with Mock, TRIM21-FL, TRIM21-△RING plasmid by Wound healing experiments. (**E)** Representative images of Brdu-positive immunofluorescence staining for transfected cells in GBM cells (U87-MG and U251-MG) transfected with Mock, TRIM21-FL, and TRIM21-ΔRING plasmid. Scar bar, 100 μm. Data are presented as means ± SD. $**p < 0.01$, $***p<0.001$.

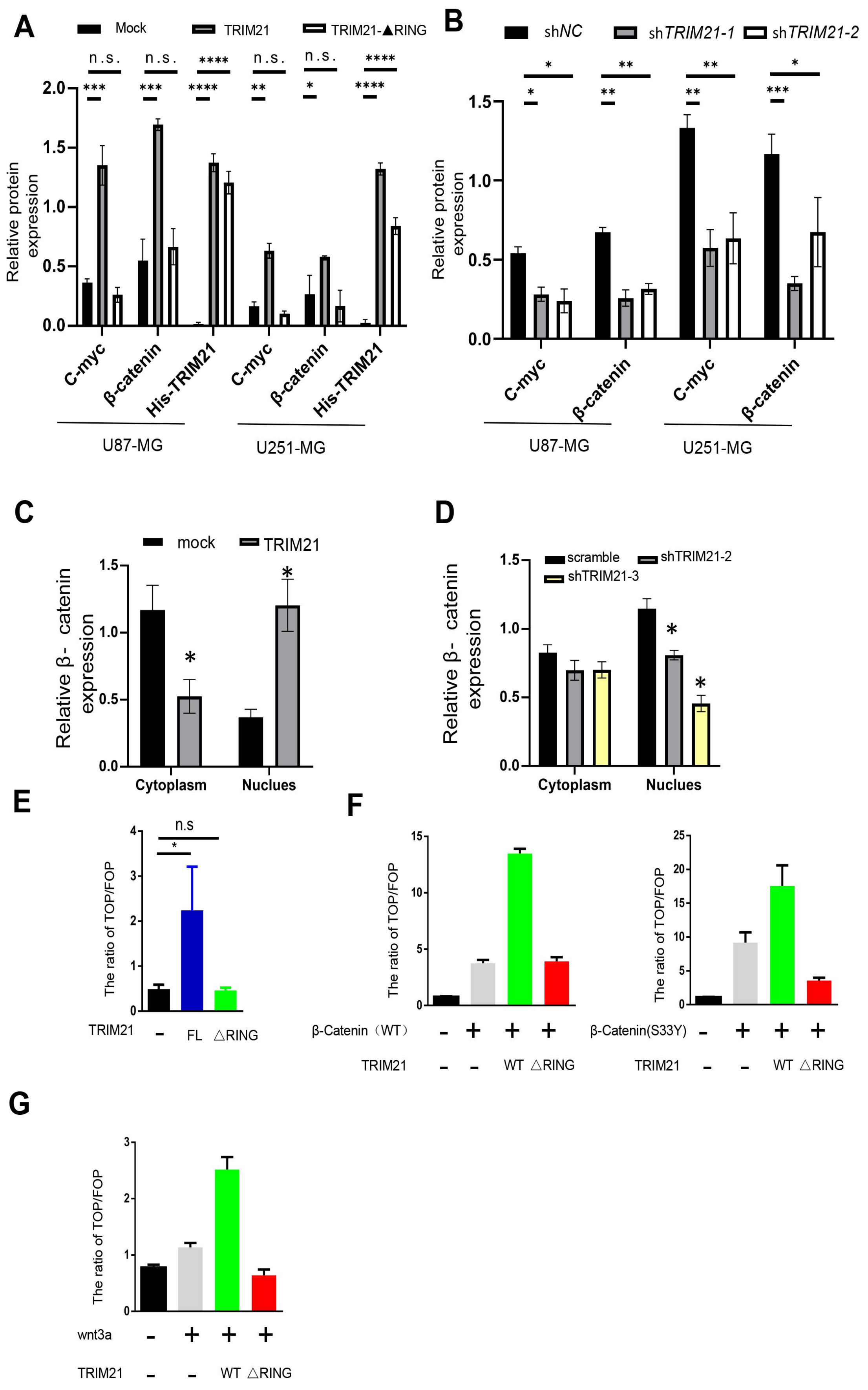
A
Mock
TRIM21
TRIM21-▲RING
Relative protein expression
C-myc
β-catenin
His-TRIM21
U87-MG
U251-MG
B
shNC
shTRIM21-1
shTRIM21-2
Relative protein expression
C-myc
β-catenin
U87-MG
U251-MG
C
mock
TRIM21
Relative β- catenin expression
Cytoplasm
Nuclues
D
scramble
shTRIM21-2
shTRIM21-3
Relative β- catenin expression
Cytoplasm
Nuclues
E
The ratio of TOP/FOP
TRIM21
FL
△RING
F
The ratio of TOP/FOP
β-Catenin（WT）
TRIM21
WT
△RING
β-Catenin(S33Y)
G
The ratio of TOP/FOP
wnt3a
TRIM21
WT
△RING

Supplementary Figure 3: TRIM21-mediated WNT/β-catenin is GSK-3β or WNT independent

(A) Quantification of β-catenin, C-Myc TRIM21 in Mock and OE-TRIM21 OETRIM21-ΔRING U87-MG and U251-MG cells. (**B)** Quantification of β-catenin, C-Myc in scramble and shTRIM21-2 shTRIM21-3 U87-MG (**C)** The β-catenin from nucleus and cytoplasm in mock and OE TRIM21 cell respectively. (**D)** Quantification of β-catenin from nucleus and cytoplasm in scramble and sh*TRIM21-2* and sh*TRIM21-3*-GBM cell respectively. **(E)** the ratios of TOP/FOP assay were shown in U87-MG transfected with the indicated plasmids. Luciferase activity was measured 36h after transfection by the dual-luciferase assay. Luciferase activity ratio is shown as relative TOP-luciferase activity compared with that in cells transfected with the FOP-luciferase activity. (**F)** The ratio of TOP/FOP assay were shown in U87-MG were transfected with the indicated plasmids. Luciferase activity ratio is shown as relative TOP-luciferase activity compared with that in cells transfected with the FOP-luciferase activity. (**G)** The ratio of TOP/FOP assay were measured. U87 cells transfected with or without TRIM21 were transfected with TOP-FLASH or FOP-FLASH, which was followed by WNT3a treatment for 10h. Data are presented as means ± SD. $**p < 0.01$, $***p<0.001$.

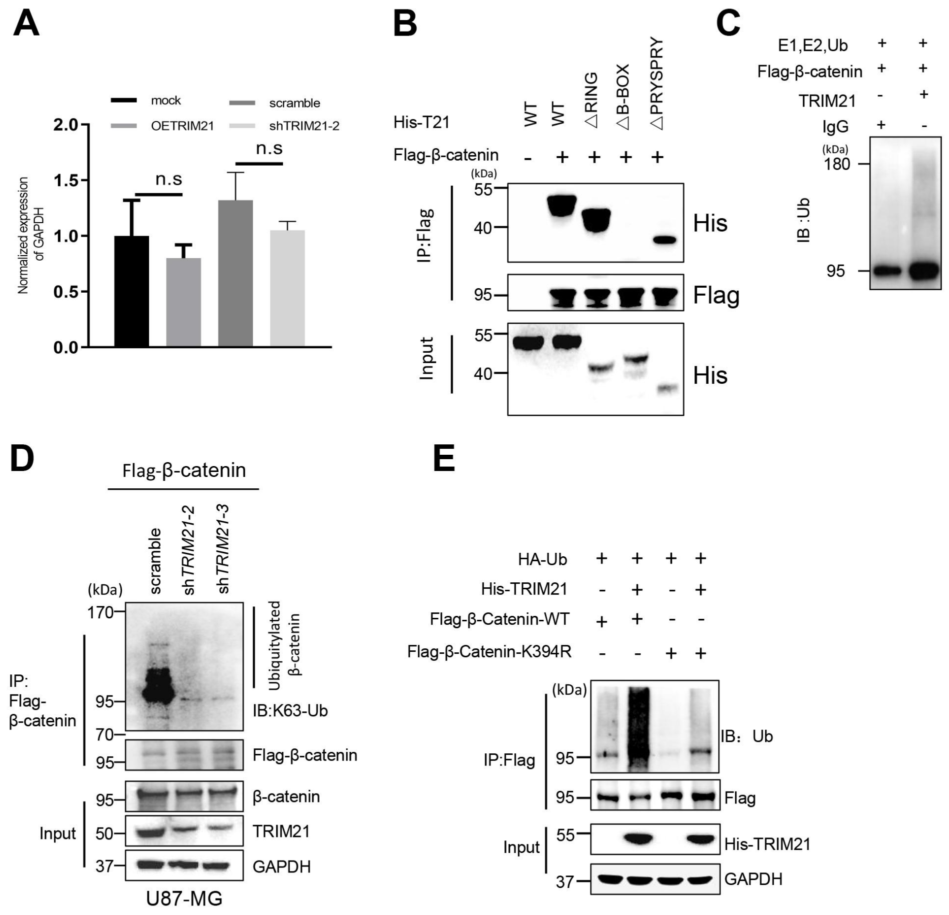

Supplementary Figure 4: TRIM21 ubiquitylates β-catenin

(A) The mRNA of β-catenin in OETRIM21 or shTRIM21-2 as compared with control cells. (**B)** 293 T cells were co-transfected with Flag-β-catenin and WT His-TRIM21 His-TRIM21-△RING， HA-TRIM21-△BOX， HA-TRIM21-△PRYSPRY domains for 48 h. CO-IP assay was performed. (**C)** In vitro ubiquitylation assays were performed as described in Materials and Methods, and the ubiquitylated form of β-catenin was detected by immunoblotting using an anti-ubiquitin antibody. (**D)** *In vivo* ubiquitination assays performed in U87-MG cells with or without TRIM21 depletion by shRNA, which were transfected as indicated. (**E)** HEK293-T cells were

cotransfected with the indicated plasmids for 48 h and then treated with 10 mM MG132. The polyubiquitination levels of Flag-β-catenin and Flag-β-catenin-K394R mutant protein were analyzed.

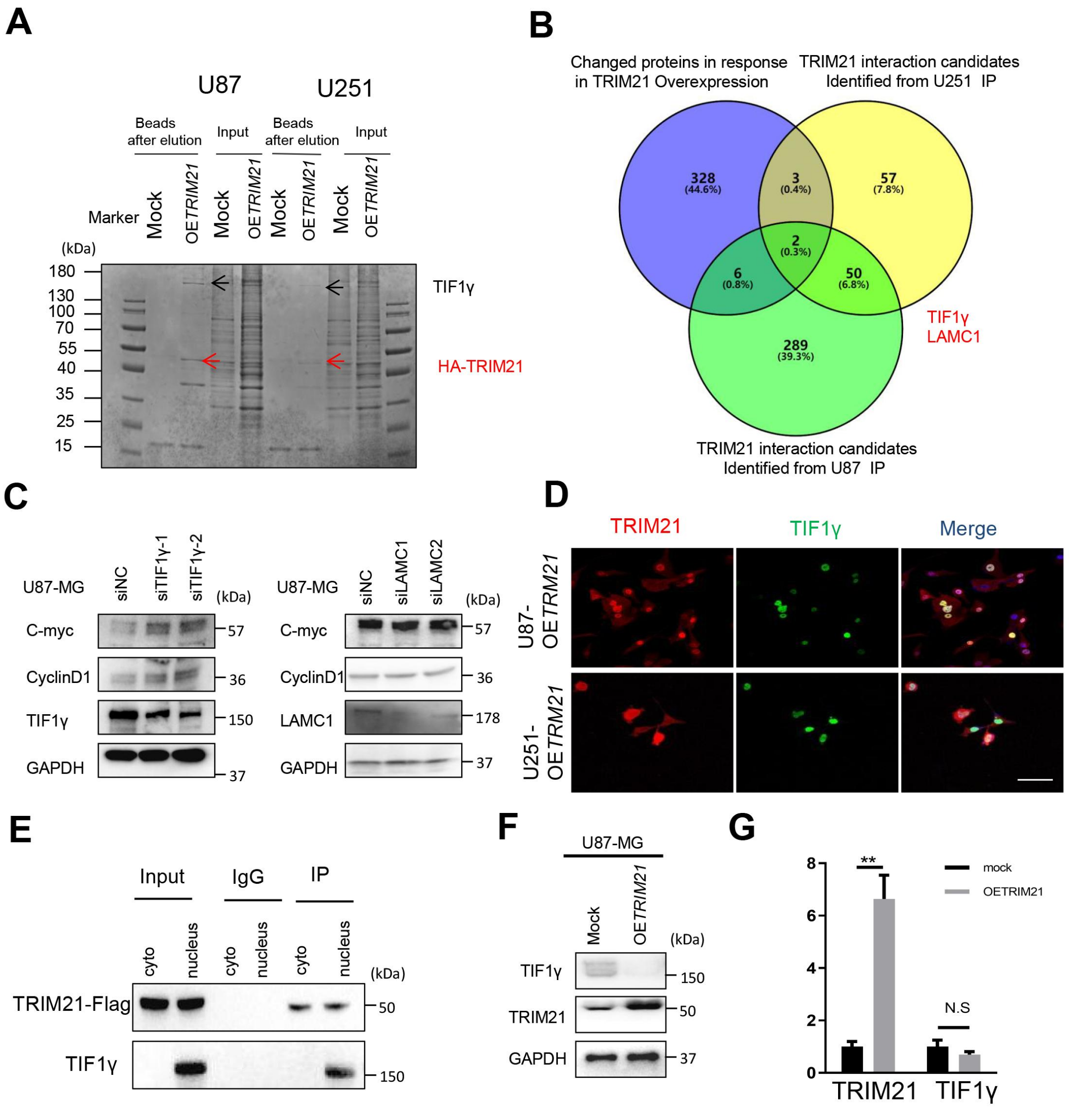

Supplementary Figure 5: Identification of potential TRIM21 substrates. **(A)** The immunoprecipitation of Flag-TRIM21 interacting protein was visualized by Coomassie blue staining. (**B)** Venn diagram showing the number of changed proteins identified in response to TRIM21 overexpression (Blue), TRIM21 interaction candidates identified in Co-IP process in U87(Green) and U251(Yellow) and overlapping proteins in the

datasets. (**C)** Expression of CyclinD1 and C-myc in U87-MG cells transfected with si*NC* or si*TIF1γ* (Left panel); si*NC* or si*LAMC1 (Right panel)* respectively. (**D)** Confocal immunofluorescence microscopy analysis of the TRIM21/TIF1γ proteins interaction in the GBM-OE*TRIM21* cells. Scarbars, 20μm. **(E)** Immunoprecipitation was performed with U87-MG-OE*TRIM21*-Flag lysates from cytoplasm and nuclues using Flag antibodies, and the precipitants were measured by western blotting with the indicated antibodies.**(F-G)** The protein **(F)** and mRNA **(G)** of TRIM21 and TIF1γ in U87-MG cells with stable transfection of control vector (Mock) or TRIM21.

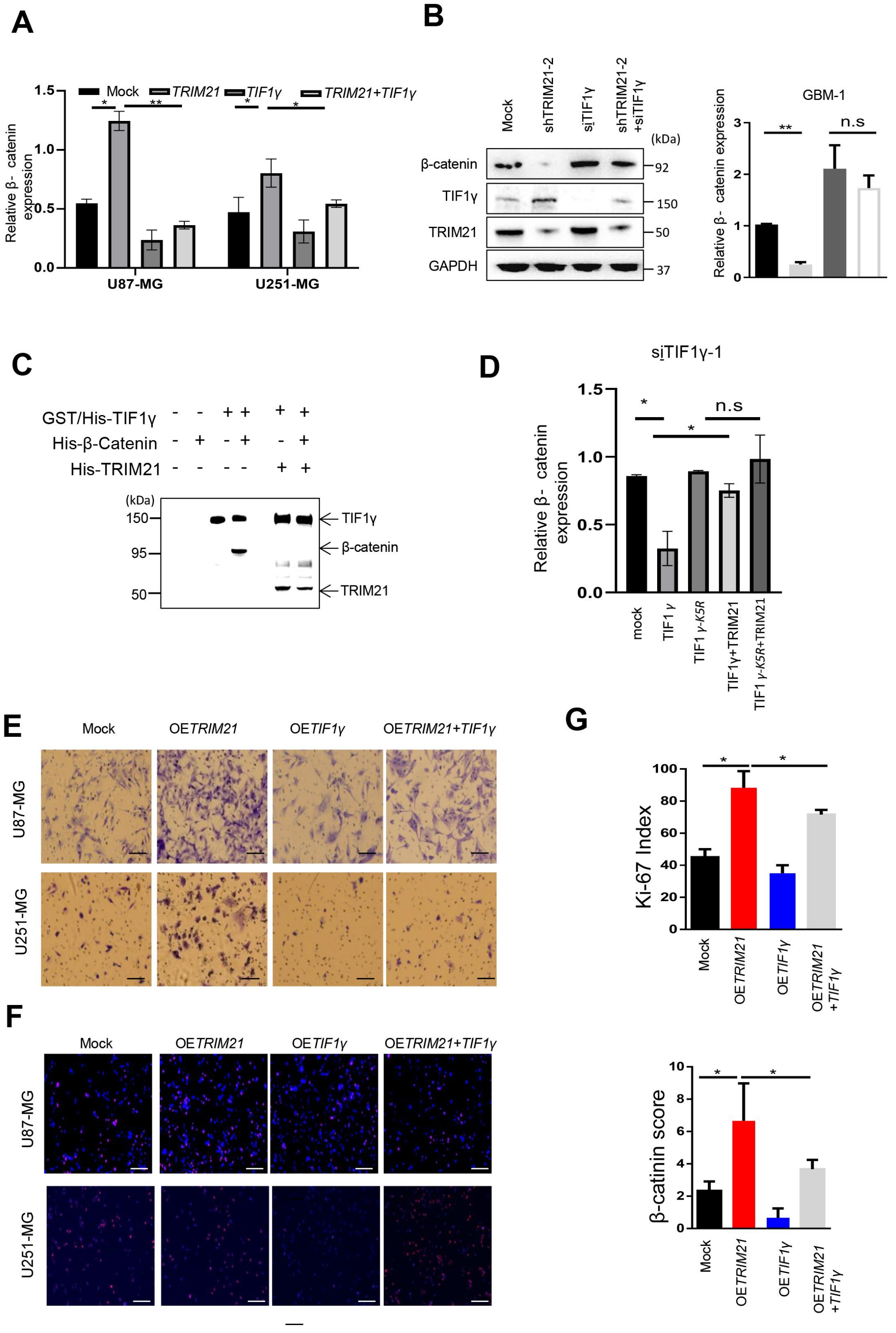
A
Mock
TRIM21
TIF1γ
TRIM21+TIF1γ
Relative β- catenin expression
U87-MG
U251-MG
B
Mock
shTRIM21-2
siTIF1γ
shTRIM21-2 +siTIF1γ
(kDa)
β-catenin
TIF1γ
TRIM21
GAPDH
92
150
50
37
GBM-1
Relative β- catenin expression
n.s
C
GST/His-TIF1γ
His-β-Catenin
His-TRIM21
(kDa)
150
95
50
TIF1γ
β-catenin
TRIM21
D
siTIF1γ-1
Relative β- catenin expression
n.s
mock
TIF1 γ
TIF1 γ-K5R
TIF1γ+TRIM21
TIF1 γ-K5R+TRIM21
E
Mock
OETRIM21
OETIF1γ
OETRIM21+TIF1γ
U87-MG
U251-MG
G
Ki-67 Index
Mock
OETRIM21
OETIF1γ
OETRIM21 +TIF1γ
F
Mock
OETRIM21
OETIF1γ
OETRIM21+TIF1γ
U87-MG
U251-MG
β-catinin score
Mock
OETRIM21
OETIF1γ
OETRIM21 +TIF1γ

Supplementary Figure 6: TRIM21/TIF1γ axis was involved in progression of GBM.

(A) Quantification of β-catenin, in Mock, OE-TRIM21 OE-TIF1γ OETRIM21+TIF1γ in U87-MG and U251-MG cells. (**B)** Immunoblotting of TRIM21, TIF1γ and β-catenin in GBM cells (U87-MG and U251-MG) knockdown TRIM21, TIF1γ and TRIM21 + TIF1γ **(C)** In vitro GST-pull-down assay showing the binding ability of GST-tagged full-length TIF1γ, His-β-catenin and His-TRIM21. The asterisk shows an unspecific protein band. (**D)** Quantification of β-catenin in U87-siTIF1γ cells transfected with indicated plasmid. (**E)** The invasion of Mock, OE*TRIM21*, OE*TIF1γ* and OE*TRIM21+ TIF1γ* GBM Cells (U87-MG and U251-MG). The data was collected after 24h of incubation. Scar bar, 50μm (**F)** Representative images of Brdu-positive immunofluorescence staining for the indicated GBM cells. Scar bars, 50μm. (**G)** The ki67 index and β-catenin score of xengaft from Mock, OE*TRIM21*, OE*TIF1γ* and OE*TRIM21+ TIF1γ* GBM Cells. Data are presented as means ± SD. $^{**}p < 0.01$, $^{***}p<0.001$.

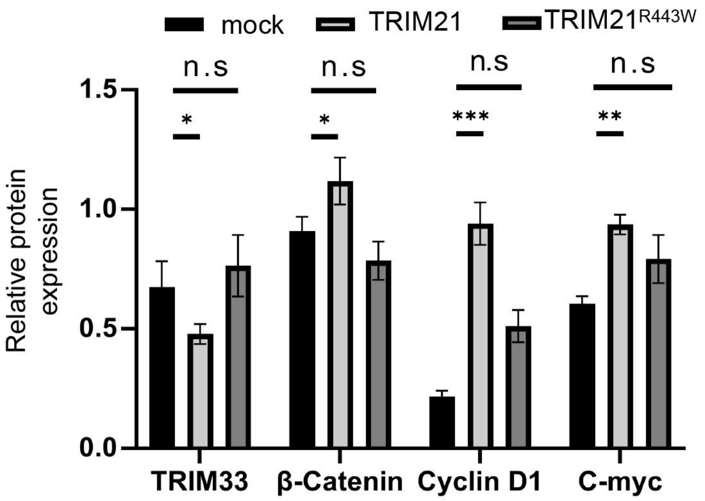

Supplementary Figure 7: Quantification of β-catenin, c-myc and cyclinD1 TRIM21 in Mock-, OE-TRIM21- OETRIM21-R443W- U87-MG respectively

Supplementary Table 1 The clinical features of the glioma specimens used in this study.

Feature	Number (Percentage)
Gender	
Male	77(64.17%)
Female	43(35.83%)
Age (year, median ± SD)	43.25±12.80
IDH1 status	
WT	77(64.17%)
MT	43(35.83%)
Predominant lobe of tumor location	
Frontal	33 (27.5%)
Temporal	42 (35.0%)
Parietal	9 (7.5%)
others	36 (30%)
Ki67	
＞=20%	20 (87.07%)
＜20%	100 (12.93%)
Grade	
I	13
II	54
III	35
IV	18

Abbreviation: WT：wildtype；MT：Mutation

Supplementary Table 2 The potential ubiquitylation sites of TIF1γ are shown. The possibility of ubiquitylation of lysine residues in TIF1γ (middle panel) was predicted by analyses of the amino acid sequence of TIF1γ

Peptide	position	Score	Threshold
***MAENKGGGEAES	5	2.28	0.30
SRREAEPKLLPCLHS	141	0.41	0.30
APSSSDEKSEQVCTS	212	1.08	0.30
HQRVKFTKDHLIRKK	252	0.49	0.30
TKDHLIRKKEDVSES	258	0.79	0.30
KDHLIRKKEDVSESV	259	0.56	0.30
PVFCPVHKQEQLKLF	280	0.44	0.30
AIENLLAKLLEKKNY	329	0.86	0.30
LLAKLLEKKNYVHFA	333	1.02	0.30
NEINKKGKSLLQQLE	378	2.13	0.30
TGLSRQVKHVMNFTN	410	0.42	0.30
STALLYSKRLITFQL	432	1.31	0.30
SSGRTAEKTSLSFKS	763	2.16	0.30
EKTSLSFKSDQVKVK	769	2.23	0.30
SFKSDQVKVKQEPGT	774	0.31	0.30
KSDQVKVKQEPGTED	776	0.81	0.30
CSFSGGVKQEKTEDG	793	1.69	0.30
SGGVKQEKTEDGRRS	796	1.90	0.30
ADMNESCKQSGLSSL	850	1.95	0.30
LSSLVNGKSPIRSLM	861	0.54	0.30
LCCEKCPKVFHLTCH	907	1.29	0.30
CDNLQHSKKGKTAQG	950	0.65	0.30
DNLQHSKKGKTAQGL	951	1.08	0.30
LQHSKKGKTAQGLSP	953	1.73	0.30
PMDLSTVKKKLQKKH	1007	1.62	0.30
MDLSTVKKKLQKKHS	1008	1.32	0.30
DLSTVKKKLQKKHSQ	1009	1.73	0.30
TVKKKLQKKHSQHYQ	1012	1.51	0.30
VKKKLQKKHSQHYQI	1013	0.64	0.30
SEVAQAGKAVALYFE	1067	2.13	0.30
DFIQPRRKRLKSDER	1115	3.03	0.30
QPRRKRLKSDERPVH	1118	2.22	0.30
DERPVHIK*******	1127	3.94	0.30
